# A novel approach to combat *Pseudomonas aeruginosa*: repurposing pharmaceuticals for inhibition of phospholipase A

**DOI:** 10.1128/spectrum.01304-25

**Published:** 2026-01-21

**Authors:** Matea Modric, Rocco Gentile, Raphael Moll, Ifey Alio, Wolfgang R. Streit, Karl-Erich Jaeger, Holger Gohlke, Filip Kovacic

**Affiliations:** 1 Forschungszentrum Jülich GmbH, Institute of Molecular Enzyme Technology, Heinrich Heine University Düsseldorf 9170, Düsseldorf, Germany; 2 Institute for Pharmaceutical and Medicinal Chemistry, Heinrich Heine University Düsseldorf 9170, Düsseldorf, Germany; 3 Department of Microbiology and Biotechnology, University of Hamburg 14915, Hamburg, Germany; 4 Forschungszentrum Jülich GmbH, Institute of Bio- and Geosciences (IBG-4: Bioinformatics) 28334, Jülich, Germany; Ross University School of Veterinary Medicine, Basseterre, Saint Kitts and Nevis

**Keywords:** intracellular bacterial phospholipases, antibiotic potentiation, molecular dynamics, inhibition, imipenem, rilapladib, darapladib, GW4869, antivirulence drugs, *Pseudomonas aeruginosa*, phospholipase A

## Abstract

Phospholipase A (PLA) plays critical roles in cellular physiology, making human PLAs established drug targets. On the other hand, the potential of bacterial PLAs as targets for antimicrobial drug development remains underexplored. In this study, we curated a library of 23 approved and investigational pharmaceuticals, some of which inhibit human PLA-like enzymes, through a combination of ligand structure-based searches and textual mining in literature and compound databases. Experimental screening identified that compounds GW4869, darapladib, and rilapladib significantly inhibit *Pseudomonas aeruginosa* growth by more than 50%. While these compounds did not reduce biofilm formation, GW4869 increased the proportion of dead cells in established biofilms, suggesting its role in compromising biofilm cell viability. Biochemical assays revealed that all three compounds inhibited the enzymatic activity of PlaF, a PLA virulence factor of *P. aeruginosa*, by decreasing the affinity of a model substrate. Molecular dynamics simulations and binding free energy analyses indicate that GW4869 binds to the substrate-binding and product-release tunnels of PlaF, suggesting GW4869 as a non-covalent competitive inhibitor. Notably, the mutant strain *P. aeruginosa* Δ*plaF* proved to be GW4869 resistant and did not display differential growth upon GW4869 treatment, further indicating PlaF as the primary GW4869 target. Furthermore, GW4869 and rilapladib significantly enhanced the efficacy of the last-resort antibiotic imipenem in combination treatments. Additionally, rilapladib exhibited broad-spectrum antibacterial activity by inhibiting the growth of both *Escherichia coli* and *Staphylococcus aureus*, while several other pharmaceuticals demonstrated species-specific effects, highlighting their potential for targeted antimicrobial applications beyond *P. aeruginosa*. These findings highlight the potential of GW4869, darapladib, and rilapladib to act as repurposed inhibitors of PlaF or PLA-dependent mechanisms in bacterial pathogens and underscore the promise of combination therapies against intracellular PLAs to combat antimicrobial resistance.

**IMPORTANCE:**

This study explores how existing drugs could be repurposed to fight *Pseudomonas aeruginosa*, a hospital-associated bacterial pathogen notorious for its strong antimicrobial resistance. By targeting intracellular phospholipase A, which are key to maintaining membrane balance, these drugs, originally developed for non-infectious diseases, may provide a fresh approach to tackling infections that are becoming harder to treat with standard antibiotics. The findings not only highlight the potential of phospholipases as promising antimicrobial targets but also uncover unexpected ways human drugs can interact with bacterial physiology. One standout compound, a preclinically studied drug called GW4869, both slows bacterial growth and boosts the effectiveness of the last-resort antibiotic imipenem, suggesting better outcomes with combination treatments. Overall, this research points to the exciting possibility of repurposing human-focused medicines as new antimicrobial agents to help combat the escalating crisis of antibiotic resistance while deepening our insight into how these drugs can influence microbes.

## INTRODUCTION

*Pseudomonas aeruginosa* is a nosocomial gram-negative pathogen that poses a significant threat to public health, particularly among immunocompromised patients (1, 2). Its multidrug resistance has led the World Health Organization to classify it as a priority pathogen requiring the urgent development of effective antibiotics and alternative treatment strategies (3, 4). The overuse and misuse of antibiotics, inadequate waste management, and rapid environmental transmission have further exacerbated antibiotic resistance in *P. aeruginosa* and other ESKAPE pathogens (5, 6). To address the global resistance crisis that hinders the effectiveness of existing antibiotics against *P. aeruginosa* (7), recent advances have been made in the development of innovative antivirulence approaches against this human pathogen (5, 8, 9).

These antivirulence compounds target key virulence determinants, reducing pathogenicity or enhancing susceptibility to antibiotics, thereby improving infection treatment outcomes (9–13). Among them, the most promising approaches include the inhibition of quorum sensing (14, 15), surface-associated adhesins (16), and iron acquisition pathways (17) that collectively contribute to biofilm formation (18), inhibition of secretory systems (19), and direct neutralization of extracellular toxins such as phospholipase A (PLA) (20). However, monotherapy with these compounds has shown limited success in eradicating infections (18, 21). Combination therapies, such as quorum-sensing inhibitors and tobramycin or the iron chelator deferoxamine and tobramycin (22), have demonstrated enhanced efficacy in eliminating *P. aeruginosa* biofilms in infected hosts. These findings underscore the potential of adjuvant therapies targeting virulence factors to become integral components of clinical practice (8, 23, 24).

Among the virulence factors of *P. aeruginosa*, secreted PLAs play an important role in host cell invasion, membrane disruption, and immune evasion by releasing fatty acids from host phospholipids (25, 26). Notably, pseudolipasin A, a selective inhibitor of the ExoU phospholipase, has shown protective effects *in vitro* on hamsters, amoebae, and yeast cells against ExoU-mediated toxicity (20). Beyond secreted phospholipases, emerging evidence reveals the significance of intracellular bacterial phospholipases for virulence (27, 28) and antibiotic resistance (29). Their function is related to the adjustment of the bacterial membrane phospholipid composition to adapt to environmental stimuli. For example, *Brucella melitensis* PLA BveA increases the resistance to polymyxin B through the hydrolysis of membrane phosphatidylethanolamine (29). Similarly, the intracellular PLA PlaF of *P. aeruginosa* regulates membrane phospholipid composition, thereby influencing iron uptake, motility, biofilm formation, and signaling pathways (28). PlaF-deficient strains exhibit reduced virulence in mouse macrophage and *Galleria mellonella* infection models, underscoring their therapeutic potential (27, 30).

The crystal structure of PlaF reveals a transmembrane helix anchoring the catalytic domain, which contains a serine–hydrolase catalytic triad, to the cytoplasmic membrane (27). This configuration facilitates the interaction of PlaF with membrane phospholipid substrates and enables substrate extraction via one of three distinct active-site tunnels (31, 32). The crystallized complex of PlaF with the fatty acid product and molecular dynamics (MD) simulations identified putative product egress tunnels (31, 32). Detailed structural and mechanistic insights furthermore emphasize the drugability of PlaF.

In light of the challenges posed by traditional drug discovery, this study explores the repurposing of Food and Drug Administration (FDA)-approved or clinically tested compounds as potential inhibitors of PlaF. Some of the selected compounds have been described to target the human serine hydrolase family PLAs (33). From a library of 23 identified compounds, we experimentally tested their effects on *P. aeruginosa* PA01 growth, biofilm formation, and dispersal. Growth-inhibiting compounds were further assessed for PlaF inhibition using functional enzyme assays and a Δ*plaF* mutant strain. Enzyme kinetics and molecular dynamics simulations indicate the mode of action of the PlaF-targeting compound GW4869. Experiments to mimic combination therapy demonstrated synergism between GW4869 and the last-resort antibiotic imipenem, supporting the therapeutic potential of PlaF as an antivirulence target. This study underscores the inhibition of intracellular PLAs as a novel and previously underexplored strategy for developing effective treatments against *P. aeruginosa*.

## MATERIALS AND METHODS

### Protein expression and purification

Expression and purification of PlaF were performed as described previously (34). *P. aeruginosa* PA01 cells were transformed (35) with plasmid pBBR-*plaF_H6_* or pBBR-*plaF_H6_*-F229W (36) and grown overnight at 37°C in lysogeny broth (LB) medium supplemented with tetracycline (100 µg/mL). These cultures were used to inoculate an expression culture in LB medium to an initial optical density at 600 nm (OD_600nm_) of 0.05. The cultures were grown at 37°C until an OD_600nm_ of ~2 was obtained and afterward harvested by centrifugation at 6,750 × *g* and at 4°C for 15 min. The total membrane fraction was solubilized with Triton X-100, and proteins were purified using Ni-NTA agarose (Qiagen, Hilden, Germany) with buffer supplemented with 0.25 mM *n*-dodecyl-β-D-maltoside. For biochemical analysis, proteins were transferred to Tris-HCl buffer (100 mM, pH 8) supplemented with the respective detergent. The purity of PlaF was analyzed by sodium dodecyl sulfate-polyacrylamide gel electrophoresis under denaturation conditions on a 12% (wt/vol) gel (37).

### Selection and preparation of compounds for screening library

The compound library was assembled based on the 2D structural similarity to six approved pharmaceuticals identified through a literature search, as indicated in Table 1. The DrugBank database was queried with these six selected compounds, and structures with more than 50% 2D structural similarity to the initial compounds were included in the library. Additionally, the Selleckchem database was searched using the keyword “phospholipase,” and the phospholipase inhibitors were added to the library. The stock solutions of selected compounds were prepared using dimethyl sulfoxide, methanol, or water as indicated in Table S1.

**TABLE 1:** Library of pharmaceuticals^*a*^

	Compound	Clinical status^*b*^*^,^*^*c*^	Human target	Mode of action	Literature
Approved drugs	Rivastigmine	Approved by FDA and EMA	Acetylcholinesterase, butyrylcholinesterase	Pseudo-irreversible inhibitor	(33, 38, 39)
	Donepezil	Approved by FDA and EMA	Acetylcholinesterase	Reversible competitive inhibitor	(33, 38)
	Tacrine	Approved by FDA	Acetylcholinesterase, cholinesterase	Non-competitive reversible inhibitor	(33, 38, 40)
	Clofazimine	Approved	Phospholipase A2 Bacterial respiratory chain Potassium channel, Wnt–β-catenin pathway	Agonist modulator inhibitor	(41–43)
	Galantamine	Approved by FDA and EMA	Acetylcholinesterase, cholinesterase, nicotinic acetylcholine receptor	Reversible competitive inhibitor	(33, 38)
	Orlistat	Approved by FDA and EMA	Triacylglycerol lipase	Competitive reversible inhibitor	(33, 44, 45)
DrugBank	Tetrabenazine	Approved	Synaptic vesicular amine transporter	Reversible inhibitor	(46, 47)
	Codeine	Approved	Opioid receptors	Agonist, regulator	(48, 49)
	Frovatriptan	Approved	5-Hydroxytryptamine receptor	Agonist	(50)
	Perindopril	Approved	Angiotensin-converting enzyme	Inhibitor	(51, 52)
	Pancuronium	approved	Muscle nicotinic acetylcholine receptor	Competitive inhibitor	(53, 54)
	Vecuronium	Approved	Muscle nicotinic acetylcholine receptor	Competitive inhibitor	(54)
	Cantharidin	Approved	Protein phosphatase	Inhibitor	(55)
	Rilapladib	Investigational	Lipoprotein-associated phospholipase A2	Inhibitor	(56)
Selleckchem	Darapladib	Investigational	Lipoprotein-associated phospholipase A2	Competitive inhibitor	(57–59)
	Quinacrine	Investigational	Cytosolic phospholipase A2, DNA	Inhibitor intercalation	(60–62)
	Varespladib	Investigational	Secretory phospholipase A2	Inhibitor	(63, 64)
	RHC80267	Experimental	Diacylglycerol lipase, cholinesterase, cyclooxygenase, phospholipases C and A2	Inhibitor	(65–67)
	ML348	Experimental	Acyl protein thioesterase 1, lysophospholipase A1	Reversible inhibitor	(68–70)
	Cambinol	Experimental	Neutral sphingomyelinase 2, sirtuin 1/sirtuin 2	Uncompetitive inhibitor	(71–73)
	GW4869	Investigational	Neutral sphingomyelinase 2	Non-competitive inhibitor	(74–76)
	Tanshinone I	Investigational	Phospholipase A2, histone lysine methyltransferase, prostaglandin E2, tumor necrosis factor-α	Inhibitor	(77–80)
	Polydatin	Investigational	Phospholipase A2, NF-κB and MAPK pathway, COX-2, iNOS, SIRT1	Inhibitor modulator	(81–83)

^*a*^ The data are sourced from DrugBank or Selleckchem databases.

^*b*^ Information was obtained from official websites of the U.S. FDA and the EMA.

^*c*^ EMA, European Medicines Agency; FDA, Food and Drug Administration.

### Growth curves

The growth of bacterial strains *P. aeruginosa* PA01 wild type, an isogenic mutant Δ*plaF* (27), *Staphylococcus aureus* ATCC 25923, and *Escherichia coli* ATCC 25922 was monitored by measuring the OD_600nm_ for 10 h in a 96-well plate shaken with agitation at 1,000 rpm. First, the OD_600nm_ of overnight cultures was diluted with lysogeny broth medium to 0.05. When the bacterial cultures reached an OD_600nm_ of approximately 0.4, 1.5 µL of inhibitor stock solutions (Table S1), 1.5 µL of antibiotic, or both were added in a total volume of 150 µL. The antibiotics were prepared in water at the following final concentrations: 0.5 mg/L (0.70 µM) for gentamicin, 2.0 mg/L (3.71 µM) for piperacillin, 1.0 mg/L (0.71 µM) for colistin, and 2.0 mg/L (6.30 µM) for imipenem. Cultures treated with respective solvents were used as no-compound controls.

### Enzyme activity assay, inhibition, and enzyme kinetic studies

The esterase activity of the PlaF protein was determined spectrophotometrically with *p*-nitrophenyl butyrate (*p*-NPB) as a substrate, as described previously (84), using a 96-well microplate. Five microliters of purified protein (stock concentration 30 µg/mL) was mixed with 2 µL of potential inhibitor stock solution at concentrations of: 1.73 mM for GW4869, 3.34 mM for codeine, and 10.0 mM for all the remaining compounds, and 93 µL of freshly prepared 1 mM *p*-NPB solution was added. Esterase activity was monitored by an increase in absorbance at 420 nm for up to 2 h at 37°C. Kinetic parameters were determined by measuring activity with different substrate concentrations (0.05, 0.1, 0.2, 0.3, 0.5, 1.0, 1.3, and 1.5 mM) (27). Half-maximal inhibitory concentration (IC_50_) was determined by measuring enzyme activity with different compound concentrations and with 1 mM *p*-NPB substrate and was calculated from linear plots (85).

### Biofilm assay

The microtiter dish biofilm assay was performed according to a modified protocol (86, 87). The respective number of single colonies of *P. aeruginosa* was inoculated in LB media and incubated for 24 h at 37°C with shaking. Overnight cultures were used to inoculate 150 µL of bacterial cultures of OD_600_ = 0.05 in LB medium in a plastic 96-well microtiter plate (MTP). In the case of compound-treated biofilm, 2 µL of the compound or respective solvent was added to a total volume of 150 µL. The inoculated microtiter plate was covered with air-permeable sealing film and incubated for 24 h at 37°C. Afterward, the OD_600nm_ was measured; the medium was removed; and each well was briefly washed two times with 200 µL LB media at 37°C. Biofilms were stained by adding 200 µL 0.1% (wt/vol) crystal violet solution, followed by 20 minutes of incubation at room temperature. The crystal violet solution was discarded, and the plate was washed three times with 200 µL water, followed by adding 200 µL 30% (vol/vol) acetic acid. The plate was incubated for 20 minutes at room temperature, and absorbance was measured at 585 nm using a plate reader (SpectraMax iD3; Molecular Devices GmbH, München, Germany).

### Colony-forming unit count

Biofilm was grown as described above. After 24 h incubation, the supernatant was discarded; the biofilm was suspended in 200 µL LB medium, diluted 100,000-fold, and 100 µL of cell suspension was plated on LB medium agar plates. Plates were incubated overnight at 37°C.

### Quantification of extracellular DNA in biofilm

Extracellular DNA was quantified according to a modified protocol (88). Biofilm was grown in a 96-well plate as described above. After washing two times with 200 µL LB medium, 100 µL of TE buffer (10 mM Tris-HCl, 1 mM EDTA, pH 8) was added, followed by adding 100 µL of SYBR Green I (Thermo Fisher Scientific, Germany). SYBR Green I was prepared freshly by diluting it 1:1,250 in TE buffer. The plate was incubated for 10 minutes in the dark at room temperature, and the fluorescence was measured using a fluorescence plate reader (SpectraMax iD3, Molecular Devices GmbH) at 485/518 nm excitation/emission filter setup.

### Confocal laser scanning microscopy of biofilm

Confocal laser scanning microscopy (CLSM) of biofilm was performed as described previously (89). Briefly, bacterial biofilms were prepared under static conditions in an eight-well chamber µ-slide (ibidi GmbH, Gräfelfing, Germany) and treated with the compounds listed in Table S1. After 24 h incubation, the samples were incubated with live/dead staining solution (LIVE/DEAD BacLight Bacterial Viability Kit; Thermo Fisher Scientific, Waltham, USA) to assess the viability of the bacterial cells. The biofilms were visualized by Confocal Laser Scanning Microscope 800 (Axio observer.Z1/7; Carl Zeiss AG, Oberkochen, Germany) at settings listed in Table S2.

### Preparation of starting structures for unbiased molecular dynamics simulations

The crystal structure of the PlaF dimer (PDB ID 6I8W) is available in the Protein Data Bank (90). The last five residues of the C-terminus of each monomer missing in the structure were added using MODELLER (91), and all small molecule ligands were removed. The dimer was oriented in the membrane using the PPM server (92). From that, the monomeric configuration of PlaF chain A was generated by removing chain B from the dimer orientation. Chain A was oriented again using the PPM server, resulting in the tilted configuration t-PlaF.

The structure of GW4869 was prepared starting from its canonical SMILES using the fixpka option in OpenEye (93). The most favorable conformer was generated with Omega 4.1.1.1 (94) using the flag –maxconfs = 1. The charges of GW4869 were calculated following the RESP approach (95) using Gaussian 16 to compute electrostatic potentials at the HF/6-31G* level (96). GW4869 docking to t-PlaF was performed using AutoDock 3.0.5 (97) with the in-house developed scoring function DrugScore (98). The selected configuration represents the most populated cluster obtained.

The bound t-PlaF configuration was embedded into a DOPE:DOPG = 3:1 membrane (99) and solvated using PACKMOL-Memgen (100, 101). The membrane composition resembled that of the native inner membrane of gram-negative bacteria (99) and was already used for simulating PlaF in a membrane bilayer environment (27, 31, 32). A distance of at least 15 Å between the protein or membrane and the solvent box boundaries was kept. To obtain a neutral system, counter ions were added that replaced solvent molecules (KCl 0.15 M), resulting in the systems containing ~140,000 atoms.

### Unbiased molecular dynamics simulations of selected inhibitors bound to t-PlaF

The GPU particle mesh Ewald implementation from the AMBER23 suite of molecular simulation programs (102) with the ff14SB (103), Lipid21 (104), and GAFF2 (105) force fields for the protein, membrane lipids, and ligands, respectively, was used; water molecules and ions were parametrized using the TIP3P model (106) and the Li and Merz 12-6 ion parameters (107, 108). For each t-Plaf-inhibitor complex, five independent replicas of 1 μs length were performed. Covalent bonds to hydrogens were constrained with the SHAKE algorithm (109) in all simulations, allowing the use of a time step of 2 fs. Details of the thermalization of the simulation systems are given below.

All unbiased MD simulations showed structurally invariant protein structures and membrane phases evidenced by electron density calculations (Fig. S1). The root mean square deviation (RMSD) of GW4869, after removal of the global motions of t-PlaF, showed structurally invariant binding configurations across five different replicas (Fig. S2).

### Relaxation, thermalization, and production runs

An initial minimization step was performed with the CPU code of pmemd (110). Each minimization was organized in four steps of 1,000 cycles each, for a total of 4,000 cycles of minimization. Afterward, each minimized system was thermalized in one stage from 0 to 300 K over 25 ps using the NVT ensemble and the Langevin thermostat (111), and the density was adapted to 1.0 g/cm^3^ over 4,975 ps using the NPT ensemble with a semi-isotropic Berendsen barostat (112), with the pressure set to 1 bar. Thermalization and density adaptation were performed with the GPU code of pmemd (110).

For each replica, 1 μs of production run using the GPU code of pmemd was performed in the NPT ensemble at a temperature of 300 K using the Langevin thermostat (111) and a collision frequency of 1 ps^−1^. To avoid noticeable distortions in the simulation box size, semi-isotropic pressure scaling was employed using the Berendsen barostat (112) with a pressure relaxation time of 1 ps, coupling box size changes along the membrane plane (113).

### Molecular mechanics–Poisson Boltzmann surface area calculations of GW4869 binding to t-PlaF

To pinpoint the most likely binding epitopes, we generated 3D density grids to map the location of GW4869 across multiple replicas. We considered stably bound conformations if a frame of the trajectory has a ligand RMSD of <1.5 Å to the previous frame (see supplementary results). These binding configurations were clustered using the hierarchical agglomerative (bottom-up) algorithm implemented in cpptraj (114), using the minimum distance *ε* between the clusters as the cluster criterion. Starting from *ε* = 2.0 Å, we gradually increased *ε* in 0.5 Å intervals until the population of the largest cluster remained unchanged (at *ε* = 4.0 Å). We calculated the 3D density maps of GW4869 considering all atoms using the grid function available in cpptraj (114) with a grid spacing of 1.5 Å. We applied a contour level of 1*σ* (one standard deviation above the mean value).

Subsequently, we conducted molecular mechanics–Poisson Boltzmann surface area (MM-PBSA) calculations with MMPBSA.py (115) and normal mode analysis computations to determine the binding effective energy and the binding entropy of GW4869 to t-PlaF, respectively. These calculations were performed across the different replicas, and the results per replica were averaged. Dielectric constants of 1 for the protein, 80 for the solvent, and 15 for the membrane were used, similar to our previous work (32). A heterogeneous dielectric model was used to represent the membrane; the implicit membrane model using spline fitting (memopt = 3) was employed for the binding effective energy calculations (116). This allowed us to compute Δ*G*_solv_ and Δ*G*_gas+solv_ (equations 1 and 2) (117). The normal mode analysis calculation allowed us to calculate the loss of configurational entropy upon binding, *−T*Δ*S*_total_, considering the translational, rotational, and vibrational terms (equation 3) (117).


(1) $$\Delta G_{solv}=\;\Delta G_{PB}+\Delta G_{np}$$

(2) $$\Delta G_{gas+solv}=\;\Delta H_{gas}+\Delta G_{solv}$$

(3) $$\Delta G_{bind}=\;\Delta G_{gas+solv}-T\Delta S_{total}$$

To compare the computed with experimentally determined binding affinities, we converted $\Delta G_{bind}$ into the standard free energy of binding $\Delta G_{bind}^{0}$ according to equation 4, as done previously (118, 119). This takes into account that translational entropy depends on solute concentration (120, 121), leading to the concentration dependence of chemical equilibria that do not conserve the number of molecules (such as binding reactions) (122, 123):


(4) $$\Delta G_{bind}^{0}=\;\Delta G_{bind}+RTln\frac{C^{ideal}}{C^{0}}$$

where *R* is the universal gas constant (*R* = 0.001987 kcal/K/mol); *T* = 298.15 K; *C*^0^ is the standard concentration of 1 mol/L; and *C*^ideal^ is the ligand concentration of 0.041 mol/L, derived from the general gas equation at a pressure of 101,325 Pa and a temperature of 298.15 K (118). $\Delta G_{bind}^{0}$ is directly related to the computed dissociation constant $K_{D}^{comp}$ according to equation 5:


(5) $$\Delta G_{bind}^{0}=RTln\left(K_{D}^{comp}\right)$$

The total standard error of the mean of the computations, denoted as ${SEM}_{total}$, is estimated following the principles of Gaussian error propagation according to equation 6:


(6) $$SEM_{total}=\;\sqrt{{\left(SEM_{G_{eff}}\right)}^{2}+{\left(SEM_{TS}\right)}^{2}},$$

where ${SEM}_{G_{eff}}$ and ${SEM}_{TS}$ are the SEMs from MM-PBSA and NMA computations, respectively. The results from binding free energy calculations are reported as $\Delta G_{bind}^{0}\;\pm \;{SEM}_{total}$. The computations were converged, as evidenced by the comparison between the first and second halves of the trajectories (Fig. S3).

## RESULTS

### Identifying pharmaceuticals with potential inhibitory effects on bacterial PLA

This study aimed to identify approved drugs and pharmaceuticals from (pre)clinical studies that have potential inhibitory activity against the bacterial phospholipase PlaF to evaluate their suitability for repurposing as antimicrobials. In the following, we will jointly refer to these compounds as pharmaceuticals. According to the literature, the six FDA-approved drugs, rivastigmine (33, 38), donepezil (33, 38), galantamine (33, 38), tacrine (33, 38), orlistat (33, 44), and clofazimine (41, 42), target human PLAs or esterases/lipases.

Despite the generally low sequence identity between human and bacterial PLAs, both enzyme families share a conserved catalytic Ser–Asp dyad and a hydrophobic substrate-binding pocket that accommodates phospholipid acyl chains, enzymes known for their side PLA activity due to the shared serine hydrolase catalytic mechanism (27, 36, 124, 125). This conservation in the catalytic mechanism provides a mechanistic rationale for exploring human PLA or serine hydrolase inhibitors as potential scaffolds for bacterial PLA inhibition. At the same time, differences in the sequence similarity between PlaF, the human α/β hydrolase domain (ABHD6), and two epoxide hydrolases identified as the most similar targets in humans (Fig. S4) were considered favorable to identify selective bacterial PLA inhibition. Indeed, structural models of the target sequences superimposed with PlaF (Fig. S5) revealed, even when unaligned residues are not considered, a high RMSD for O60906; in all other cases, it is around 2 Å or below (Table S3). Still, when analyzing the binding site residues, we identified that a high proportion of residues were in alignment gaps (Table S3) and that the binding site cavity of PlaF slightly overlaps only with ABHD6 but not in the other structures, suggesting high structural diversity (Fig. S5). To accommodate this, we therefore enlarged the diversity in the screening pool by adding other compounds based on structural similarity.

**Fig 1:**
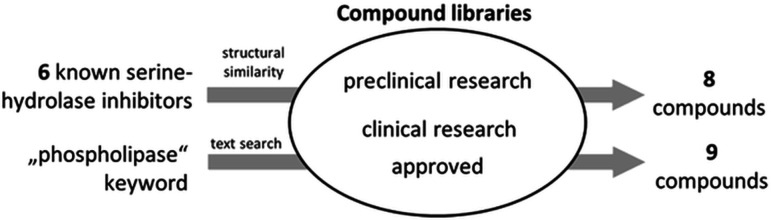
Selection of potential phospholipase PlaF inhibitors. A library of 23 pharmaceuticals to be tested as potential PlaF inhibitors (Table 1) was generated by screening compounds in preclinical and clinical research phases and approved drugs, from compound libraries of DrugBank and Selleckchem databases. The selection criteria involved a combination of structural similarity and text-based searches.

Identified pharmaceuticals were used as templates to identify similar compounds among more than 500,000 investigational, clinically tested, or approved drugs in the DrugBank database (126, 127). This ligand-based search identified eight additional compounds showing 2D structural similarity with the initial six compounds, with similarity scores ranging from 54% to 72%. Furthermore, a targeted keyword search in the Selleckchem database, which includes over 120,000 bioactive small molecules, yielded nine additional compounds being investigated as human phospholipase, lysophospholipase, or lipase inhibitors (Fig. 1).

Of the resulting 23 pharmaceuticals, 13 have regulatory approval, 7 are in clinical research phases, and 3 are in preclinical studies, target esterases, lipases, and phospholipases. For the cases of tetrabenazine, codeine, frovatriptan, perindopril, pancuronium, vecuronium, and cantharidin, no related target has been described, despite an apparent molecular similarity to the template compounds or associated keywords. The advantage of this library is that the majority of the compounds have met safety and efficacy standards for use in humans (Table 1). In conclusion, this library of diverse potential phospholipase-inhibiting compounds provided a foundation for testing their repurposing as antimicrobial agents.

### Effect of potential PlaF inhibitors on the growth and biofilm formation of *P. aeruginosa*

**Fig 2:**
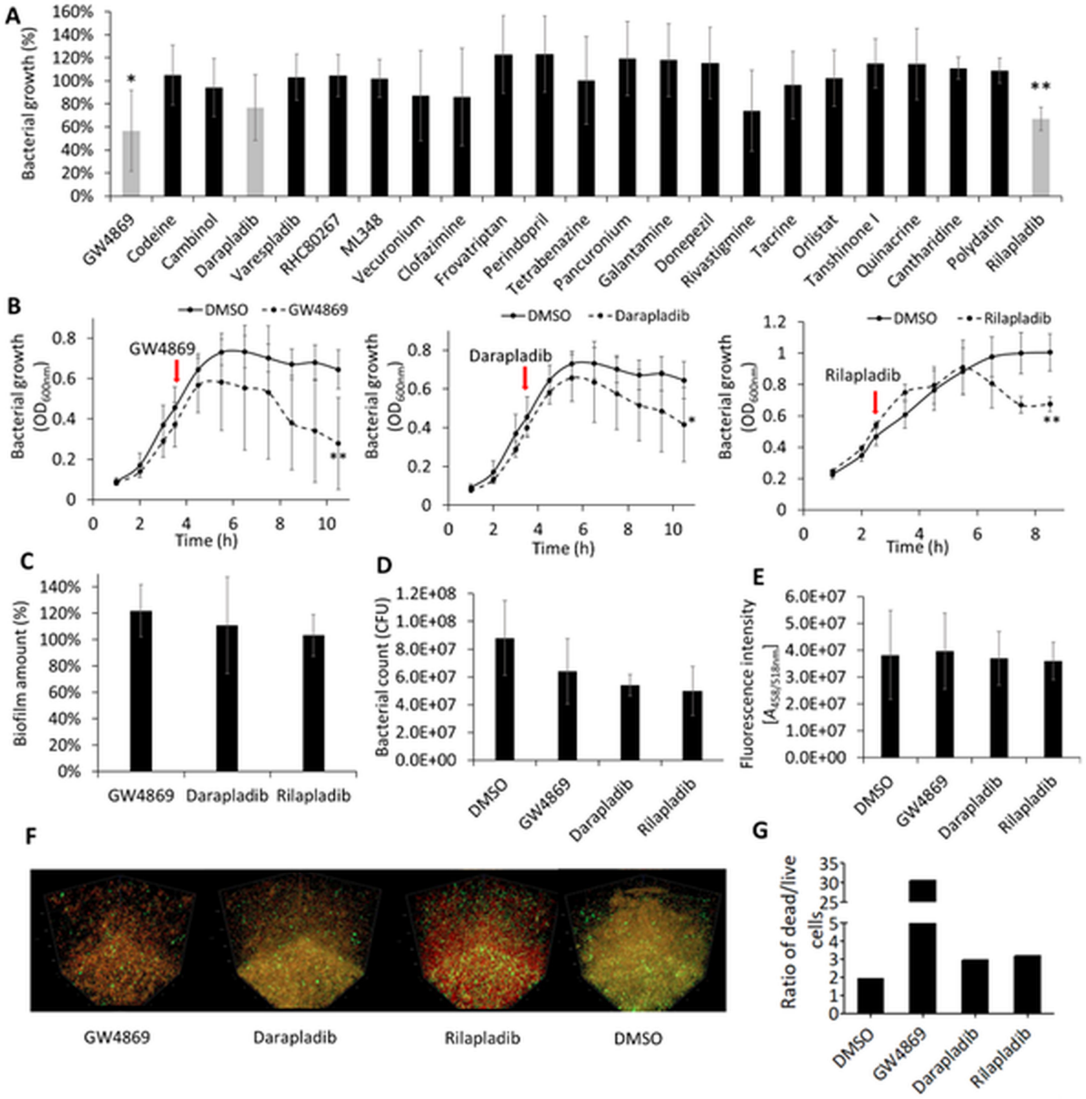
Potential PLA inhibitors impact the growth and viability of *P. aeruginosa* PA01. (**A**) Effect of 23 pharmaceuticals on the planktonic growth of *P. aeruginosa* after 5 h incubation with compounds. Relative growth values represent optical densities (OD_600nm_) of compound-treated cultures compared to the OD_600nm_ of respective solvent-treated cultures set to 100%. Light gray bars represent pharmaceuticals that significantly impacted the growth of *P. aeruginosa* after 5 h or more (see panel **B**). Black bars represent compounds that showed no significant effect on the *P. aeruginosa* growth at any time point. Results are shown as the mean ± SD of six biological replicates (*n* = 6) from two independent experiments. *t*-test of normally distributed values; *P < 0.05, **P < 0.01. (**B**) Growth curves of *P. aeruginosa* treated with GW4869 (17.3 µM), darapladib (100.0 µM), and rilapladib (100.0 µM). Bacterial cultures were grown in LB medium at 37°C with shaking at 1,000 rpm in a plastic MTP. Red arrows indicate the beginning of treatment. Results are the mean ± SD of three independent experiments (*n* = 3). *t*-test of normally distributed values; *P < 0.05, **P < 0.01. (**C**) Biofilm amount of *P. aeruginosa* treated for 24 h with GW4869 (17.3 µM), darapladib (100.0 µM), and rilapladib (100.0 µM) was quantified by the crystal violet assay. The results are shown relative to the solvent-treated cultures, which were set to 100%. The results are the mean ± SD of >22 biological replicates from four independent experiments. *t*-test was calculated compared to the untreated samples. (**D**) Viable bacterial count determined as colony-forming units (CFUs) within biofilm treated for 24 h with GW4869 (17.3 µM), darapladib (100.0 µM), and rilapladib (100.0 µM) or the respective solvent (dimethyl sulfoxide [DMSO]). The *y*-axis represents colony-forming units per 1 mL of bacterial culture. Results are the mean ± SD of four biological replicates (*n* = 4); *t*-test showed no significant changes between treated and untreated cultures. (**E**) Quantification of eDNA in biofilm treated for 24 h with GW4869 (17.3 µM), darapladib (100.0 µM), and rilapladib (100.0 µM) or the respective solvent (DMSO) determined with SYBR Green I dye. Results are the mean ± SD of four biological replicates (*n* = 4); *t*-test showed no significant changes between treated and untreated cultures. (**F**) Representative confocal scanning microscopy figures of *P. aeruginosa* PA01 grown as static biofilms treated for 24 h with GW4869 (7.5 µM), darapladib (100.0 µM), rilapladib (100.0 µM), or DMSO as the solvent control. Biofilms were stained to visualize live (green) or dead/dying (red) cells. (**G**) The ratio of dead to live cells in biofilms was determined from red and green fluorescence intensity quantification from panel **F** using the BiofilmQ tool.

We investigated the effects of the 23 pharmaceutical compounds from the library on the planktonic growth of *P. aeruginosa* in a rich LB medium. In a screening experiment designed to identify the most potent pharmaceuticals, the compounds were added to exponentially growing bacterial cultures (OD_600nm_ ~0.5) in plastic MTPs, and optical density was measured after incubation at 37°C with agitation. In our study, relatively high concentrations of pharmaceuticals were used to overcome bioavailability issues due to poor aqueous solubility of many compounds. The results demonstrated that GW4869 and rilapladib significantly inhibited bacterial growth compared to the respective solvent-treated controls after 5 h incubation, while the effect of darapladib is significant after 7 h (Fig. 2A). Validation growth inhibition experiments under these screening conditions revealed that the effects of all three compounds were evident as early as 2–4 h post-treatment (Fig. 2B). Over 6–7 h, treated cultures exhibited a slower growth rate compared to the controls. After 6–7 h, darapladib and rilapladib reduced *P. aeruginosa* growth significantly by approximately 35%, while GW4869 exhibited a more pronounced effect, reducing growth by 57% (Fig. 2B).

Additionally, evaluating the pharmaceutical compounds from our library, we found that rilapladib inhibited the growth of gram-negative enteropathogenic *E. coli* ATCC 25922 by 44% after 6 h of treatment (Fig. S6). Furthermore, the growth of pathogenic gram-positive *S. aureus* ATCC 25923 was reduced by 58% and 73% following treatment with rilapladib and darapladib, respectively (Fig. S7). Several other pharmaceuticals from the library also show specificity to inhibit the growth of *E. coli* and *S. aureus* in the range of 30%–55% (Fig. S6 and S7).

Biofilm formation represents a dominant lifestyle of *P. aeruginosa* during chronic infections (128, 129), functioning as a protective barrier against antibiotics (130, 131). Therefore, we next assessed the effects of GW4869, darapladib, and rilapladib on biofilm formation and cell viability in static cultures (without agitation) adhered to plastic MTP. Pharmaceuticals were introduced at the time of inoculation, followed by a 24-h incubation at 37°C under static conditions. Crystal violet staining of adherent cells revealed that these pharmaceuticals had no significant effect on biofilm quantity (Fig. 2C). Viable bacterial counts assessed by colony-forming unit assays indicated no significant differences between treated and untreated biofilms (Fig. 2D). Furthermore, quantification of extracellular DNA, a critical biofilm structural component linked to increased antibiotic resistance (132), showed no significant differences between the treated and untreated biofilms (Fig. 2E).

Given that the tested pharmaceuticals did not affect initial biofilm formation steps, we next investigated their impact on biofilm dispersal. Using CLSM, we examined biofilm architecture and cell viability. In this experiment, *P. aeruginosa* biofilms were preformed by incubating bacteria at 37°C under static conditions on microscopic glass slides for 24 h, followed by an additional 24 h treatment with GW4869, darapladib, or rilapladib. Biofilms were then stained with live/dead viability dyes: a green dye for live cells and a red dye for cells with compromised membranes, assigned as dead or dying cells. Results showed no discernible effect of the tested pharmaceuticals on biofilm dispersal (Fig. 2F). However, the ratio of dead/dying cells was much higher in biofilms treated with all three compounds compared to untreated controls (Fig. 2G). Quantitative analysis of CLSM images revealed that the dead-to-live cell ratio increased from 2:1 in untreated biofilms to approximately 3:1 in biofilms treated with darapladib or rilapladib. Notably, GW4869 had a strong effect, as only about 3% of the cells were estimated to be alive.

These findings highlight the potential of GW4869, darapladib, and rilapladib as inhibitors of planktonic growth and the viability of biofilm-forming *P. aeruginosa* cultures.

### Inhibitory potential of selected compounds against PlaF

**Fig 3:**
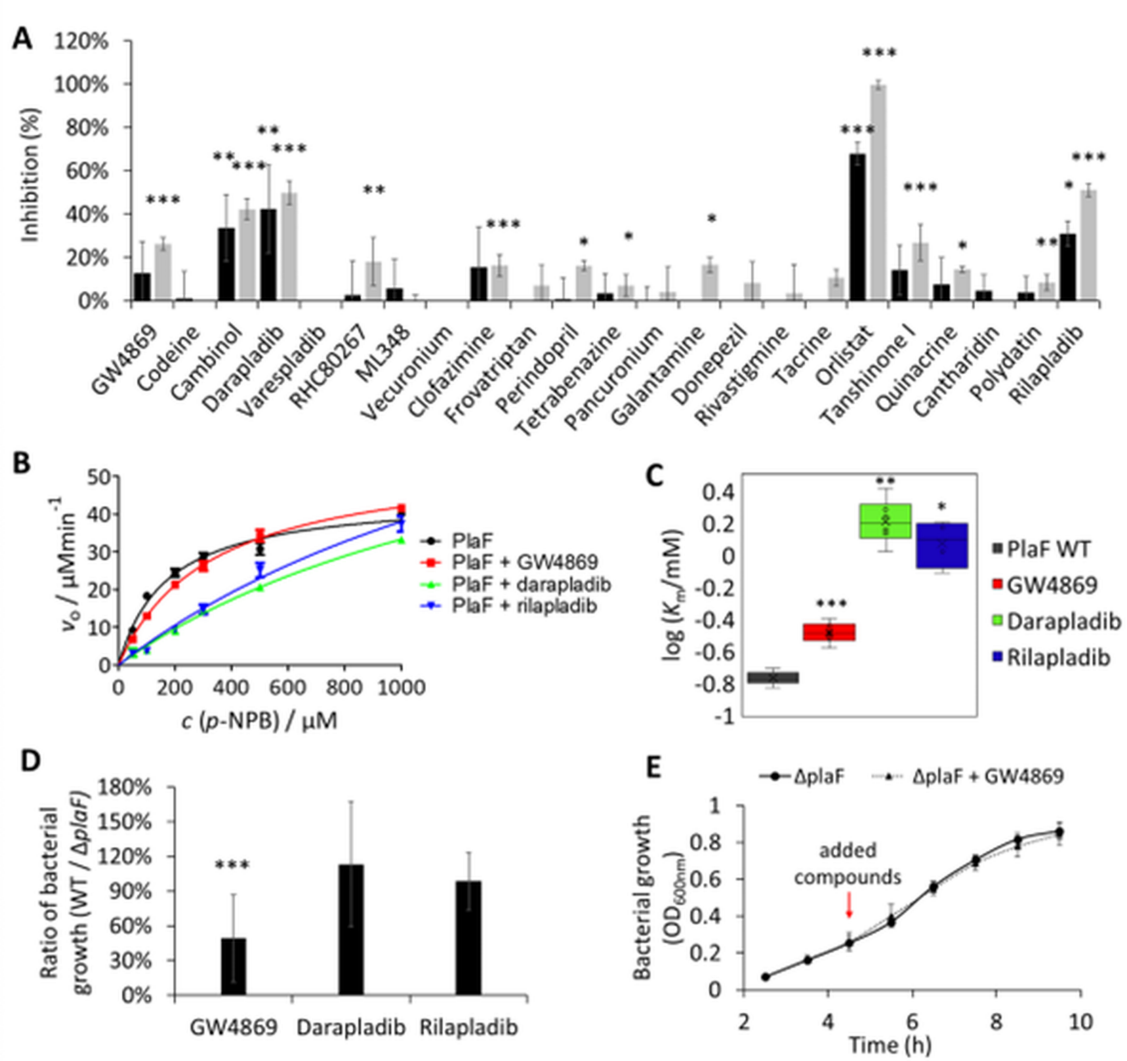
Inhibitory effect of potential PLA targeting pharmaceuticals on PlaF. (**A**) Purified PlaF was treated with compounds (34.6 and 69.2 µM for GW4869, 66.8 and 133.6 µM for codeine, and 200 and 400 µM for the remaining compounds) in the presence of the *p*-NPB substrate, and the released product was quantified spectrophotometrically during 1 h. Black and gray bars indicate lower and higher concentrations of pharmaceuticals, respectively. Results are shown as relative inhibition compared to samples treated with the respective solvent. Results are the mean ± SD of three experiments, each measured with three replicates (*n* = 9). *t*-test was calculated by comparing pharmaceutical- and solvent-treated samples; *P < 0.05, **P < 0.01, ***P < 0.001. (**B**) Michaelis–Menten curve showing enzyme kinetic studies with purified PlaF treated with GW4869 (34.6 µM), darapladib (200.0 µM), or rilapladib (200.0 µM) measured spectrophotometrically using *p*-NPB assay. The curves are calculated with GraphPad software from two independent experiments, each measured three times (*n* = 6). (**C**) Log-transformed Michaelis–Menten constants calculated from curves in panel **B**. The average value is indicated with a cross, while the center line represents the median. The box limits indicate the interquartile range, and the whiskers indicate the minimum and maximum values. *t*-test of normally distributed values is calculated compared to the non-treated sample; *P < 0.05, **P < 0.01, ***P < 0.001. (**D**) The ratio of *P. aeruginosa* WT/Δ*plaF* bacterial growth after 6 h incubation with GW4869 (17.3 µM), darapladib (100.0 µM), and rilapladib (100.0 µM). Bacterial cultures were grown in LB medium at 37°C with shaking at 1,000 rpm. Results are shown as the ratio of the mean values ± SD of two independent experiments, each measured three times (*n* = 6). *t*-test of normally distributed values, ***P < 0.001. (**E**) Growth curves of PA01 Δ*plaF* treated with GW4869 (17.3 µM). Bacterial cultures were grown in LB medium at 37°C with shaking at 1,000 rpm in a plastic MTP. Red arrows indicate time points of compound addition. Results are the mean ± SD of four biological replicates (*n* = 4).

Next, we investigated whether PlaF, an intracellular PLA recently discovered by our group, serves as a potential target of GW4869, darapladib, and rilapladib. PlaF plays a critical role in maintaining phospholipid homeostasis and impacts the pathogenicity of *P. aeruginosa* (27, 28). To address this, we first performed biochemical assays using purified PlaF reconstituted into phospholipid liposomes to ensure a near-native conformation (31). To assess the inhibition of its hydrolytic activity in the presence of the three selected pharmaceuticals, a spectrophotometric assay based on the hydrolysis of the pro-chromogenic *p*-NPB substrate revealed that all three compounds inhibited PlaF activity. Darapladib and rilapladib at 400 µM reduced PlaF activity by ~50%, while GW4869 (69.2 µM) inhibited PlaF activity by ~30% (Fig. 3A). Although some of the remaining compounds showed an inhibitory effect on the PlaF activity, we did not consider them further as they did not impact the growth of *P. aeruginosa*.

Next, we employed enzyme kinetic studies to determine whether these compounds inhibit PlaF competitively. Using the *p*-NPB assay, we measured PlaF activity at various substrate concentrations (0.05–1.0 mM) and calculated the initial velocity (*v*₀) and Michaelis–Menten constant (*K*_*m*_). Kinetic plots showed that darapladib and rilapladib strongly, and GW4869 less prominently, affected the catalytic properties of PlaF (Fig. 3B) without altering *v*_max_ but increasing the *K*_*m*_ (Fig. 3C). These results indicate that PlaF treated with these compounds exhibits reduced substrate affinity, thus suggesting competitive inhibition.

To further explore whether these pharmaceuticals target PlaF in bacteria, we compared the growth of *P. aeruginosa* wild-type and an isogenic Δ*plaF* mutant strain in the presence of the compounds (Fig. 3D). Results revealed that darapladib and rilapladib showed no differential effects on the growth of the Δ*plaF* strains, indicating that their mode of action is likely independent of PlaF. In contrast, after 6 h of incubation with GW4869, the Δ*plaF* mutant grew to a higher cell density than the wild-type strain (Fig. 3D), whereas the growth curves of GW4869-treated and GW4869-untreated Δ*plaF* did not differ (Fig. 3E). These results suggest that the action of GW4869 may be directly or indirectly related to PlaF.

Overall, our findings suggest that GW4869, darapladib, and rilapladib modulate PlaF activity *in vitro*, although a distinct inhibitory effect potentially mediated by inhibition of PlaF in bacterial culture was only observed with GW4869.

### Mechanism of inhibition of PlaF by GW4869 involves binding to the substrate-binding tunnel

**Fig 4:**
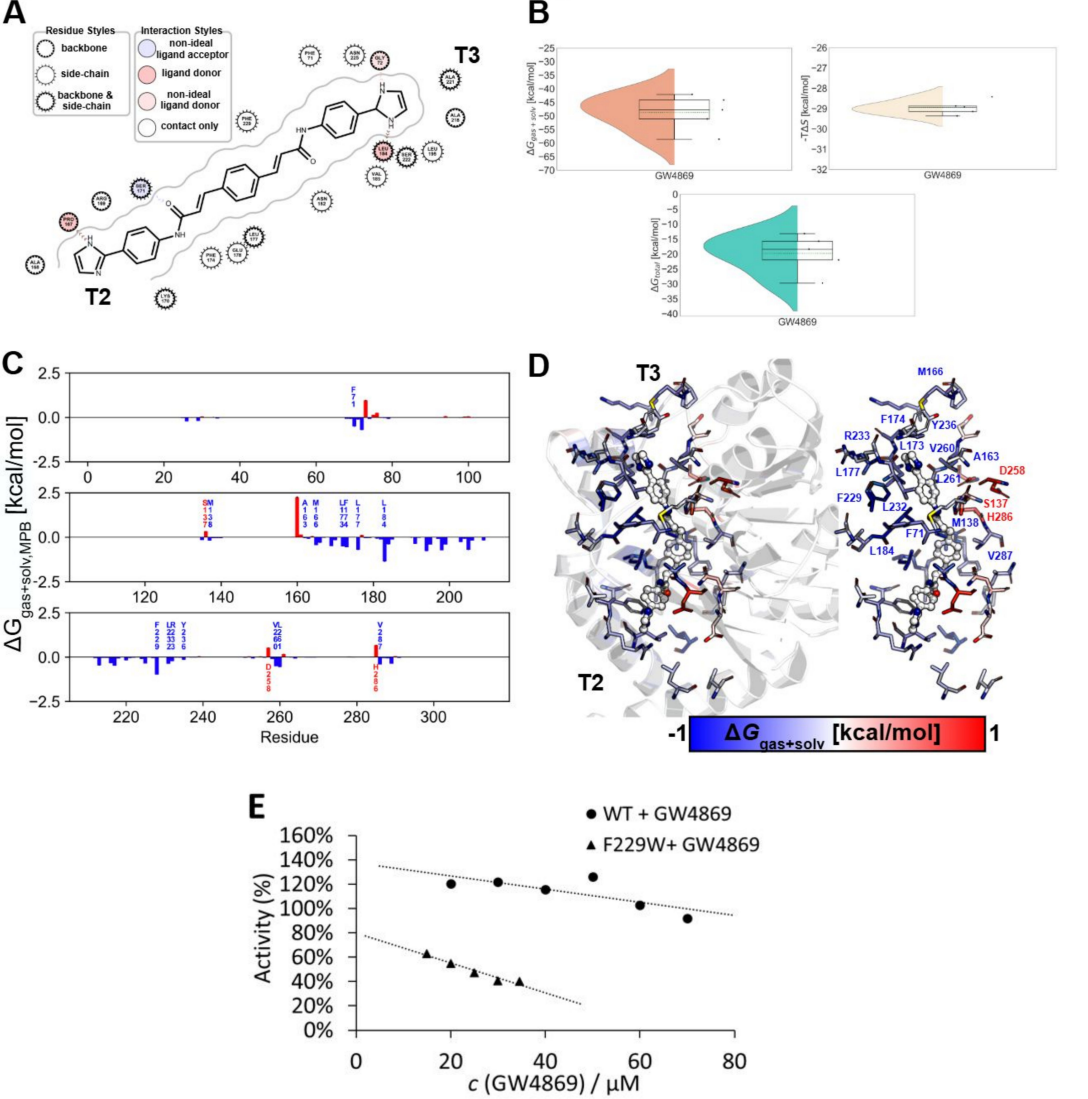
Molecular mechanism of GW4869 binding to t-PlaF. (**A**) Binding of GW4869 to t-PlaF in the most populated cluster from MD simulations represented as a 2D interaction map. Residues interact with their side chain, backbone, or both, as detailed by the legend. The entrances of tunnels T2 and T3 are indicated. (**B**) Effective energy (top left) and configurational entropy (top right) contributions to binding and total binding energy (bottom) of GW4869. The violin plots indicate the distributions of the data points; the inner box of a box plot represents the interquartile range, with the horizontal black line indicating the median. The dotted green line represents the mean, and the whiskers show the rest of the distribution, excluding points determined to be outliers when they fall outside 1.5 times the interquartile range. (**C**) Per-residue binding effective energy of tunnel residues. The energies were averaged across five independent replicas (*n* = 5). Error bars denote the SEM. (**D**) Per-residue binding effective energy mapped at the structural level. Interacting residues are depicted as sticks, colored according to the effective binding energy. The entrances of tunnels T2 and T3 are indicated. The interacting residues of T3 are labeled. The GW4869 inhibitor is depicted as red, blue, and gray spheres. (**E**) Determination of half-maximal inhibitory concentration for inhibition of phospholipid liposome-reconstituted PlaF_WT_ or PlaF_F229W_ with GW4869 using *p*-NPB assay. Graphs show linear plots of relative activities compared to the untreated sample set to 100%. The result represents the mean ± SD of six measurements (*n* = 6).

To obtain atomistic insights into the inhibition of PlaF by GW4869, we conducted unbiased MD simulations. Results from five independent 1 μs long MD simulations indicated that the t-PlaF:GW4869 complex, where the ligand was initially docked at the active site, remains structurally invariant, as indicated by a low overall RMSD of <1.5 Å (Fig. S2). Hierarchical clustering of the simulated configurations sampled every 50 ps across the five replicas revealed that GW4869 preferentially binds to tunnels T2 and T3, which are structural elements previously identified as essential for substrate hydrolysis (31, 32). The most abundant cluster (C1), where GW4869 occupies parts of T2 and T3, accounted for 25.7% ± 4.2% of the analyzed frames (Fig. 4A).

To quantify the binding energy of GW4869 in cluster C1, the unbiased MD simulations were used for MM-PBSA calculations, incorporating an implicit membrane model (115, 116). The computations were converged, as evidenced by the comparison between the first and the second halves of the trajectories (Fig. S3). These trajectories yielded a favorable binding effective energy (Δ*G*_gas+solv_) of −48.8 ± 2.9 kcal/mol and an unfavorable entropic contribution to binding (*T*Δ*S*) of −28.9 ± 0.2 kcal/mol. The resulting binding free energy (Δ*G*_bind_) estimate is −19.8 ± 2.9 kcal/mol (Fig. 4B). The corrected standard free energy of binding ($\Delta G_{bind}^{0}$) is −21.7 ± 2.9 kcal/mol, indicating that binding of GW4869 to T2 and T3 is exergonic.

The residue-specific decomposition of the binding effective energy revealed key residues in T2 and T3 that contribute significantly to GW4869 binding. Residues in T3 that were proposed to be part of the product egress pathway (31) contributed more to GW4869 binding (62.2% of the total favorable interacting residues) than residues in T2 (37.8%). This resulted in a more favorable interaction energy with T3 (Δ*G*_gas+solv, T3_ = −30.4 ± 2.2 kcal/mol) than T2 (Δ*G*_gas+solv, T2_ = −15.0 ± 3.2 kcal/mol). Notably, residue F229 in T3, previously shown to be critical for product release, exhibited one of the strongest binding interactions. Other residues near F229, including F71, L177, L184, and L232, also contributed markedly to GW4869 binding (Fig. 4C and D).

To experimentally validate the role of F229 in GW4869 binding, we aimed to determine the IC_50_ of GW4869 for wild-type PlaF (PlaF_WT_) and the F229W variant (PlaF_F229W_). Both variants were purified and reconstituted into phospholipid liposomes to ensure near-native PlaF conformations (31). Inhibition curves obtained by varying the GW4869 concentration at constant protein and substrate (*p*-NPB) concentrations displayed a linear relationship within the tested concentration range without reaching a plateau, likely due to the low solubility of the hydrophobic GW4869 in water and its adsorption to phospholipid liposomes. Although precise IC_50_ values could not be determined, the observed linear relationship suggests that GW4869 inhibits PlaF_F229W_ activity more efficiently compared to PlaF_WT_ within the tested concentration range (Fig. 4E). We speculate that the increased indolyl π-system of Trp and/or the presence of a hydrogen bond donor in the Trp ring provides more favorable interactions with GW4869 compared to the smaller phenyl ring of phenylalanine.

In summary, these findings suggest that GW4869 binds noncovalently to the active site of PlaF, which might lead to interference with substrate binding and product release.

### Compounds inhibiting *P. aeruginosa* growth potentiate the effect of common antibiotics

To evaluate the potential synergistic effects of *P. aeruginosa* growth-inhibiting pharmaceuticals GW4869, darapladib, and rilapladib (Fig. S6) with common antibiotics against *P. aeruginosa*, we conducted combinatorial treatments with antibiotics of differing modes of action (133). Synergy with antibiotics over time was assessed using growth curve analysis rather than checkerboard fractional inhibitory concentration indices (134) to keep the conditions identical to growth studies with pharmaceuticals only.

The selected last-resort antibiotics included gentamicin, which targets ribosomal function (135); colistin, which disrupts cytoplasmic membrane integrity (136); and piperacillin and imipenem, which target cell wall synthesis (137, 138). To assess synergistic effects, these bactericidal antibiotics were used at sub-inhibitory concentrations for *P. aeruginosa*. The concentrations used were as follows: gentamicin at 0.5 mg/L (0.70 µM), piperacillin at 2 mg/L (3.71 µM), colistin at 1 mg/L (0.71 µM), and imipenem at 2 mg/L (6.30 µM) (133).

**Fig 5:**
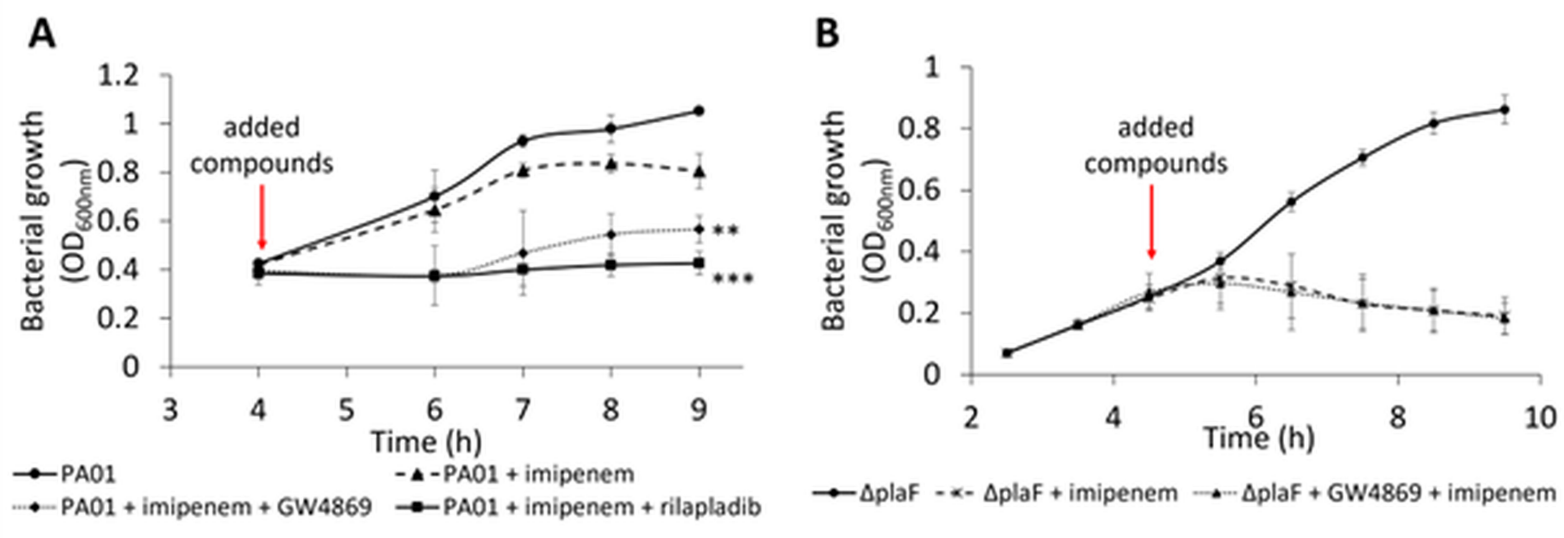
PlaF inhibitors potentiate the antibiotic activity of imipenem on *P. aeruginosa*. (**A**) The combination of imipenem (6.3 µM) and GW4869 (17.3 µM) or rilapladib (100.0 µM) resulted in reduced growth of *P. aeruginosa*. Bacterial cultures were grown in LB medium at 37°C with shaking at 1,000 rpm. Results are shown as the mean ± SD of four biological replicates (*n* = 4). *t*-test of normally distributed values; **P < 0.01, ***P < 0.001. (**B**) Growth of *P. aeruginosa* Δ*plaF* mutant in the presence of a combination of imipenem (6.3 µM) and GW4869 (17.3 µM). Bacterial cultures were grown in LB medium at 37°C with shaking at 1,000 rpm. Results are shown as the mean ± SD of four biological replicates (*n* = 4).

Treatment of planktonic *P. aeruginosa* PA01 cultures in the exponential growth phase with combinations of pharmaceutical and antibiotic revealed that darapladib did not enhance the activity of any of the four antibiotics tested (Fig. S7). Similarly, GW4869 and rilapladib did not improve the bactericidal effects of gentamicin, colistin, or piperacillin (Fig. S7). However, combinations of GW4869 or rilapladib with imipenem showed significant synergy (Fig. 5A). Imipenem at a concentration fourfold lower than its minimum inhibitory concentration had only a minimal impact on bacterial growth. In contrast, co-treatment with GW4869 (17.3 µM) or rilapladib (100 µM) abolished *P. aeruginosa* growth within 2 h and significantly decreased the optical density after an additional 3 h (Fig. 5A).

To explore the mechanism of action of GW4869, presumed to inhibit PlaF, we examined its effect in combination with imipenem on the Δ*plaF* mutant strain (Fig. 5B). Growth analyses indicated that the GW4869-imipenem combination had no different effect than imipenem alone. In both cases, growth was slowed within 1 h of treatment, with no subsequent growth over the next 4 h. These findings suggest that GW4869’s synergism with imipenem is dependent on the presence of PlaF, further supporting the hypothesis that GW4869 acts on *P. aeruginosa* by inhibiting PlaF.

## DISCUSSION

### Inhibition of intracellular PLA is a novel strategy to combat *P. aeruginosa* and other pathogens

Various virulence factors of *P. aeruginosa* play a crucial role in host infections by facilitating bacterial adhesion, invasion, immune suppression, tissue damage, and nutrient acquisition (139). These mechanisms highlight the potential of antivirulence strategies as innovative therapeutic approaches (8). This study investigates whether inhibiting intracellular phospholipase PlaF, a virulence factor of *P. aeruginosa*, can suppress the growth of bacterial culture *in vitro*, providing a foundation for developing antivirulence therapies targeting intracellular PLAs. We acknowledge that *P. aeruginosa* encodes several PLA enzymes, including ExoU, PldA, and OMPLA, which could potentially interact with the tested compounds. However, an overlay of ExoU (RCSB_ID: 3TU3), PldA (RCSB_ID: 7V53), and OMPLA-PlpD (RCSB_ID: 5FQU, full sequence AlphaFold model AF-Q9HYQ6-F1) with PlaF reveals that all structures are dissimilar from PlaF (Fig. S8). Moreover, we have explored the possibility of translating these results to pathogenic *E. coli* and *S. aureus* strains.

Intracellular PLAs are essential for remodeling the bacterial membrane phospholipid composition, a vital adaptation that enhances virulence and antibiotic resistance in *P. aeruginosa* (27, 28) and other pathogens (29). Using a target-based drug repurposing strategy, 23 potential PLA inhibitors were selected from compounds already approved or undergoing (pre)clinical trials for human diseases. This approach could leverage the safety profiles of these compounds, including data on toxicity, pharmacokinetics, and reproductive and carcinogenic effects (140). While cross-reactivity with human pathways could pose challenges, it may also offer dual benefits by targeting pathogen factors and host pathways critical for infection resolution or damage mitigation (141).

Limited aqueous solubility and poor bacterial membrane permeability likely had a negative effect on bioavailability inside bacteria, which is required for drugs acting on intracellular targets in *P. aeruginosa*. Hence, many of the tested compounds were applied at concentrations above clinically achievable levels in humans or mice, with concentrations of codeine, GW4869, cambinol, RHC80267, ML348, and varespladib being closest to a therapeutically relevant range. Therefore, to elicit antibacterial effects *in vitro*, future investigations should focus on structure–activity relationship-guided modification of the core scaffolds (142), including reducing lipophilicity to improve aqueous solubility, optimizing substituents to enhance affinity for the PlaF active-site tunnels, and incorporating polar groups to improve bacterial membrane permeability. These pathogen-directed optimization strategies aim to generate derivatives with markedly improved potency and activity at therapeutically relevant concentrations. Moreover, optimizing drug formulation by using nanocarrier-based delivery, lipid or polymeric nanoparticles, and prodrug strategies (143) could significantly enhance solubility and intracellular uptake, thereby improving effective bioavailability of poorly soluble compounds.

We have not performed cytotoxicity tests for the pharmaceuticals examined here, as these assessments had already been carried out during their respective (pre)clinical testing. However, the feasibility of repurposing these pharmaceuticals as antibacterial scaffolds remains to be elucidated through future assessment of their cytotoxicity and selectivity toward bacterial versus mammalian PLA targets. Furthermore, for clinical application, it is necessary to establish pharmacological safety profiles of optimized derivatives even if they are very similar to an approved drug.

### Efficacy of PLA inhibitors in inhibiting *P. aeruginosa*, *E. coli*, and *S. aureus* growth

Our results reveal that GW4869, darapladib, and rilapladib significantly inhibit the *in vitro* growth of planktonic *P. aeruginosa* and decrease the viability of biofilm-forming cells. Interestingly, rilapladib showed a broad inhibitory effect as it also inhibited the growth of *E. coli* and *S. aureus*. Application of putative PLA inhibitors from our library of pharmaceuticals may show potential for monotherapies, as several other inhibitors selectively inhibited the growth of pathogenic *E. coli* and *S. aureus*. The inhibitory effects of rilapladib on species that lack PlaF homologs indicate that this pharmaceutical may also target other lipid-metabolizing enzymes or membrane-associated processes. This cross-species activity suggests potential as broad-spectrum membrane disruptors and that PlaF inhibition may represent one of several contributing mechanisms.

While for rilapladib and darapladib—targeting human lipoprotein-associated phospholipase A2 (56, 57)—no clearly defined bacterial target could be identified, GW4869 demonstrated potent inhibition of the intracellular PLA PlaF. This was supported by the resistance observed in *P. aeruginosa* Δ*plaF* mutants and the inhibition of purified PlaF. PlaF is a non-essential *P. aeruginosa* gene, as deletion of *plaF* does not impair bacterial growth under standard laboratory conditions (27), suggesting that *P. aeruginosa* encodes several PlaF homologs. Such predicted single-transmembrane helix proteins (Fig. S8) could compensate for PlaF’s biological function in PL remodeling. These homologs likely reveal structural differences in their active sites, potentially preventing interactions with GW4869.

The observed competitive inhibition kinetics, consistent docking poses within the catalytic pocket, and resistance of the Δ*plaF* variant together provide indirect yet convergent evidence that GW4869 may target PlaF. However, it is likely that PlaF inhibition alone may not fully account for the observed antibacterial effects. Instead, PlaF inhibition may sensitize cells to metabolic or envelope stress, consistent with its role in membrane homeostasis. Our results suggest that GW4869’s specificity, while evident for PlaF, may not extend uniformly to all phospholipase homologs, warranting further investigation into their individual susceptibilities. While we cannot exclude contributions from other PLA-active proteins, the consistent correlation between PlaF inhibition *in vitro* and reduced bacterial growth supports PlaF as a plausible, although not exclusive, target. Although direct biophysical characterization of PlaF-ligand binding is challenging due to the high hydrophobicity of the interacting molecules, such experiments would be valuable for elucidating the mechanism of PlaF targeting by GW4869. GW4869 at 17 µM concentration, which is close to the concentration used for mice treatment (~10 µM, 5 mg/kg) (144), reduced planktonic *P. aeruginosa* growth to more than 50% within 7 h and drastically reduced biofilm-cell viability to 3% post-treatment. These findings align with prior studies showing impaired biofilm formation in *plaF* knockout strains (27). Interestingly, GW4869 had no significant impact on *Legionella pneumophila* replication or outer membrane vesicle secretion in infected human THP-1 monocytes (145), consistent with the absence of PlaF homologs in *L. pneumophila* (25).

### Synergy with antibiotics

GW4869 also demonstrated notable adjuvant activity when combined with imipenem, even at sub-minimum inhibitory concentrations. The synergy might result from reduced production of the imipenem-neutralizing β-lactamase AmpC (146) in the Δ*plaF* mutant (27). Imipenem disrupts peptidoglycan synthesis by inhibiting penicillin-binding proteins, destabilizing the cell wall (146). Thus, a dual assault on the bacterial envelope due to a putative membrane dysfunction caused by GW4869-mediated PlaF inhibition might enhance bactericidal activity.

### Potential alteration of host–pathogen interactions

Rilapladib and darapladib inhibit host lipoprotein-associated PLA2, which releases precursors of inflammatory lipid mediators (56–59). Rilapladib or darapladib would help dampen systemic inflammation and simultaneously slow bacterial proliferation, thus helping clear infections.

GW4869 is a non-competitive inhibitor of neutral sphingomyelinase 2, which regulates the conversion of sphingomyelin to ceramide (74, 75, 147). This mechanism has been shown to inhibit exosome release, which results in impaired pro-tumorigenic macrophage differentiation (148) and suppression of systemic inflammation in the murine sepsis model and LPS treatment (148). These immunomodulatory effects, coupled with its direct activity against *P. aeruginosa*, suggest that GW4869 may bolster host defenses against severe bacterial infections.

Additionally, it remains to be determined whether GW4869 mediates ceramide reduction during *P. aeruginosa* infection, as ceramide was shown to promote infection and immune evasion (149, 150).

### Future directions

Further preclinical and clinical research is required to determine whether GW4869 could be effectively used alongside antibiotics to improve outcomes in bacterial infections. GW4869 holds promise as an antivirulence compound targeting intracellular bacterial PLAs and host lipid pathways critical to host–pathogen interactions. These findings open novel avenues for the development of innovative therapies targeting intracellular PLAs to combat bacterial infections.

## SUPPLEMENTAL MATERIAL

Supplemental material spectrum.01304-25-s0001.docx Tables S1 to S3; Fig. S1 to S9.

ASM does not own the copyrights to Supplemental Material that may be linked to, or accessed through, an article. The authors have granted ASM a non-exclusive, world-wide license to publish the Supplemental Material files. Please contact the corresponding author directly for reuse.

## References

[1] Reynolds D, Kollef M. 2021. The epidemiology and pathogenesis and treatment of Pseudomonas aeruginosa infections: an update. Drugs (Abingdon Engl) 81:2117–2131. doi:10.1007/s40265-021-01635-6 PMC8572145 34743315

[2] Jesudason T. 2024. Antibacterial agents in preclinical and clinical development. The Lancet Microbe 5:100962. doi:10.1016/j.lanmic.2024.100962 39102836

[3] Murray CJL, Ikuta KS, Sharara F, Swetschinski L, Robles Aguilar G, Gray A, Han C, Bisignano C, Rao P, Wool E, et al.. 2022. Global burden of bacterial antimicrobial resistance in 2019: a systematic analysis. The Lancet 399:629–655. doi:10.1016/S0140-6736(21)02724-0 PMC8841637 35065702

[4] Melchiorri D, Rocke T, Alm RA, Cameron AM, Gigante V. 2025. Addressing urgent priorities in antibiotic development: insights from WHO 2023 antibacterial clinical pipeline analyses. The Lancet Microbe 6:100992. doi:10.1016/j.lanmic.2024.100992 39454608 PMC11876093

[5] Qin S, Xiao W, Zhou C, Pu Q, Deng X, Lan L, Liang H, Song X, Wu M. 2022. Pseudomonas aeruginosa: pathogenesis, virulence factors, antibiotic resistance, interaction with host, technology advances and emerging therapeutics. Sig Transduct Target Ther 7. doi:10.1038/s41392-022-01056-1 PMC9233671 35752612

[6] Elfadadny A, Ragab RF, AlHarbi M, Badshah F, Ibáñez-Arancibia E, Farag A, Hendawy AO, De Los Ríos-Escalante PR, Aboubakr M, Zakai SA, Nageeb WM. 2024. Antimicrobial resistance of Pseudomonas aeruginosa: navigating clinical impacts, current resistance trends, and innovations in breaking therapies. Front Microbiol 15:1374466. doi:10.3389/fmicb.2024.1374466 38646632 PMC11026690

[7] Miethke M, Pieroni M, Weber T, Brönstrup M, Hammann P, Halby L, Arimondo PB, Glaser P, Aigle B, Bode HB, et al.. 2021. Towards the sustainable discovery and development of new antibiotics. Nat Rev Chem 5:726–749. doi:10.1038/s41570-021-00313-1 PMC8374425 34426795

[8] Veesenmeyer JL, Hauser AR, Lisboa T, Rello J. 2009. Pseudomonas aeruginosa virulence and therapy: evolving translational strategies. Crit Care Med 37:1777–1786. doi:10.1097/CCM.0b013e31819ff137 19325463 PMC2749241

[9] Jiang C, Zheng L, Yan Y-J, Wang M, Liu X-J, Dai J-Y. 2024. A supramolecular antibiotic targeting drug-resistant Pseudomonas aeruginosa through the inhibition of virulence factors and activation of acquired immunity. ACS Appl Mater Interfaces 16:41828–41842. doi:10.1021/acsami.4c06665 39088848 PMC11331443

[10] Kudoh I, Wiener-Kronish JP, Hashimoto S, Pittet JF, Frank D. 1994. Exoproduct secretions of Pseudomonas aeruginosa strains influence severity of alveolar epithelial injury. American Journal of Physiology-Lung Cellular and Molecular Physiology 267:L551–L556. doi:10.1152/ajplung.1994.267.5.L551 7977765

[11] Kurahashi K, Kajikawa O, Sawa T, Ohara M, Gropper MA, Frank DW, Martin TR, Wiener-Kronish JP. 1999. Pathogenesis of septic shock in Pseudomonas aeruginosa pneumonia. J Clin Invest 104:743–750. doi:10.1172/JCI7124 10491409 PMC408437

[12] Sadikot RT, Blackwell TS, Christman JW, Prince AS. 2005. Pathogen-host interactions in Pseudomonas aeruginosa pneumonia. Am J Respir Crit Care Med 171:1209–1223. doi:10.1164/rccm.200408-1044SO 15695491 PMC2718459

[13] Kipnis E, Sawa T, Wiener-Kronish J. 2006. Targeting mechanisms of Pseudomonas aeruginosa pathogenesis. Med Mal Infect 36:78–91. doi:10.1016/j.medmal.2005.10.007 16427231

[14] Müh U, Schuster M, Heim R, Singh A, Olson ER, Greenberg EP. 2006. Novel Pseudomonas aeruginosa quorum-sensing inhibitors identified in an ultra-high-throughput screen. Antimicrob Agents Chemother 50:3674–3679. doi:10.1128/AAC.00665-06 16966394 PMC1635174

[15] Singh VK, Almpani M, Maura D, Kitao T, Ferrari L, Fontana S, Bergamini G, Calcaterra E, Pignaffo C, Negri M, de Oliveira Pereira T, Skinner F, Gkikas M, Andreotti D, Felici A, Déziel E, Lépine F, Rahme LG. 2022. Tackling recalcitrant Pseudomonas aeruginosa infections in critical illness via anti-virulence monotherapy. Nat Commun 13:5103. doi:10.1038/s41467-022-32833-9 36042245 PMC9428149

[16] Kao DJ, Churchill MEA, Irvin RT, Hodges RS. 2007. Animal protection and structural studies of a consensus sequence vaccine targeting the receptor binding domain of the type IV pilus of Pseudomonas aeruginosa. J Mol Biol 374:426–442. doi:10.1016/j.jmb.2007.09.032 17936788 PMC3493149

[17] Choi S-R, Britigan BE, Narayanasamy P. 2019. Dual inhibition of Klebsiella pneumoniae and Pseudomonas aeruginosa iron metabolism using gallium porphyrin and gallium nitrate. ACS Infect Dis 5:1559–1569. doi:10.1021/acsinfecdis.9b00100 31264851 PMC12188613

[18] Pang Z, Raudonis R, Glick BR, Lin T-J, Cheng Z. 2019. Antibiotic resistance in Pseudomonas aeruginosa: mechanisms and alternative therapeutic strategies. Biotechnol Adv 37:177–192. doi:10.1016/j.biotechadv.2018.11.013 30500353

[19] Neely AN, Holder IA, Wiener-Kronish JP, Sawa T. 2005. Passive anti-PcrV treatment protects burned mice against Pseudomonas aeruginosa challenge. Burns 31:153–158. doi:10.1016/j.burns.2004.09.002 15683685

[20] Lee VT, Pukatzki S, Sato H, Kikawada E, Kazimirova AA, Huang J, Li X, Arm JP, Frank DW, Lory S. 2007. Pseudolipasin A is a specific inhibitor for phospholipase A2 activity of Pseudomonas aeruginosa cytotoxin ExoU. Infect Immun 75:1089–1098. doi:10.1128/IAI.01184-06 17178785 PMC1828555

[21] Wang J, Lu X, Wang C, Yue Y, Wei B, Zhang H, Wang H, Chen J. 2024. Research progress on the combination of quorum-sensing inhibitors and antibiotics against bacterial resistance. Molecules 29:1674. doi:10.3390/molecules29071674 38611953 PMC11013322

[22] Moreau-Marquis S, O’Toole GA, Stanton BA. 2009. Tobramycin and FDA-approved iron chelators eliminate Pseudomonas aeruginosa biofilms on cystic fibrosis cells. Am J Respir Cell Mol Biol 41:305–313. doi:10.1165/rcmb.2008-0299OC 19168700 PMC2742750

[23] Hauser AR. 2009. The type III secretion system of Pseudomonas aeruginosa: infection by injection. Nat Rev Microbiol 7:654–665. doi:10.1038/nrmicro2199 19680249 PMC2766515

[24] Chatterjee M, Anju CP, Biswas L, Anil Kumar V, Gopi Mohan C, Biswas R. 2016. Antibiotic resistance in Pseudomonas aeruginosa and alternative therapeutic options. Int J Med Microbiol 306:48–58. doi:10.1016/j.ijmm.2015.11.004 26687205

[25] Flores-Díaz M, Monturiol-Gross L, Naylor C, Alape-Girón A, Flieger A. 2016. Bacterial sphingomyelinases and phospholipases as virulence factors. Microbiol Mol Biol Rev 80:597–628. doi:10.1128/MMBR.00082-15 27307578 PMC4981679

[26] Monturiol‐Gross L, Villalta‐Romero F, Flores‐Díaz M, Alape‐Girón A. 2021. Bacterial phospholipases C with dual activity: phosphatidylcholinesterase and sphingomyelinase. FEBS Open Bio 11:3262–3275. doi:10.1002/2211-5463.13320 PMC8634861 34709730

[27] Bleffert F, Granzin J, Caliskan M, Schott-Verdugo SN, Siebers M, Thiele B, Rahme L, Felgner S, Dörmann P, Gohlke H, Batra-Safferling R, Jaeger K-E, Kovacic F. 2022. Structural, mechanistic, and physiological insights into phospholipase A-mediated membrane phospholipid degradation in Pseudomonas aeruginosa. eLife 11:e72824. doi:10.7554/eLife.72824 35536643 PMC9132575

[28] Caliskan M, Poschmann G, Gudzuhn M, Waldera-Lupa D, Molitor R, Strunk CH, Streit WR, Jaeger K-E, Stühler K, Kovacic F. 2023. Pseudomonas aeruginosa responds to altered membrane phospholipid composition by adjusting the production of two-component systems, proteases and iron uptake proteins. Biochimica et Biophysica Acta (BBA) - Molecular and Cell Biology of Lipids 1868:159317. doi:10.1016/j.bbalip.2023.159317 37054907

[29] Kerrinnes T, Young BM, Leon C, Roux CM, Tran L, Atluri VL, Winter MG, Tsolis RM. 2015. Phospholipase A1 modulates the cell envelope phospholipid content of Brucella melitensis, contributing to polymyxin resistance and pathogenicity. Antimicrob Agents Chemother 59:6717–6724. doi:10.1128/AAC.00792-15 26282427 PMC4604402

[30] Kovacic F, Batra-Safferling R. 2025. PlaF: A bacterial lands cycle phospholipase A mediating membrane phospholipid degradation and virulence adaptation, p 387–418. Elsevier.

[31] Ahmad S, Strunk CH, Schott-Verdugo SN, Jaeger K-E, Kovacic F, Gohlke H. 2021. Substrate access mechanism in a novel membrane-bound phospholipase A of Pseudomonas aeruginosa concordant with specificity and regioselectivity. J Chem Inf Model 61:5626–5643. doi:10.1021/acs.jcim.1c00973 34748335

[32] Gentile R, Modric M, Thiele B, Jaeger K-E, Kovacic F, Schott-Verdugo S, Gohlke H. 2024. Molecular mechanisms underlying medium-chain free fatty acid-regulated activity of the phospholipase PlaF from Pseudomonas aeruginosa JACS Au 4:958–973. doi:10.1021/jacsau.3c00725 38559719 PMC10976570

[33] Bachovchin DA, Cravatt BF. 2012. The pharmacological landscape and therapeutic potential of serine hydrolases. Nat Rev Drug Discov 11:52–68. doi:10.1038/nrd3620 22212679 PMC3665514

[34] Bleffert F, Granzin J, Gohlke H, Batra-Safferling R, Jaeger K-E, Kovacic F. 2019. Pseudomonas aeruginosa esterase PA2949, a bacterial homolog of the human membrane esterase ABHD6: expression, purification and crystallization. Acta Crystallogr F Struct Biol Commun 75:270–277. doi:10.1107/S2053230X19002152 30950828 PMC6450514

[35] Choi K-H, Kumar A, Schweizer HP. 2006. A 10-min method for preparation of highly electrocompetent Pseudomonas aeruginosa cells: application for DNA fragment transfer between chromosomes and plasmid transformation. J Microbiol Methods 64:391–397. doi:10.1016/j.mimet.2005.06.001 15987659

[36] Kovacic F, Bleffert F, Caliskan M, Wilhelm S, Granzin J, Batra-Safferling R, Jaeger K-E. 2016. A membrane-bound esterase PA2949 from Pseudomonas aeruginosa is expressed and purified from Escherichia coli. FEBS Open Bio 6:484–493. doi:10.1002/2211-5463.12061 PMC4856427 27419054

[37] Laemmli UK. 1970. Cleavage of structural proteins during the assembly of the head of bacteriophage T4. Nature 227:680–685. doi:10.1038/227680a0 5432063

[38] Jann MW, Shirley KL, Small GW. 2002. Clinical pharmacokinetics and pharmacodynamics of cholinesterase inhibitors. Clin Pharmacokinet 41:719–739. doi:10.2165/00003088-200241100-00003 12162759

[39] Bono GF, Simão-Silva DP, Batistela MS, Josviak ND, Dias PFR, Nascimento GA, Souza RLR, Piovezan MR, Souza RKM, Furtado-Alle L. 2015. Butyrylcholinesterase: K variant, plasma activity, molecular forms and rivastigmine treatment in Alzheimer’s disease in a Southern Brazilian population. Neurochem Int 81:57–62. doi:10.1016/j.neuint.2014.12.009 25624079

[40] Mitra S, Muni M, Shawon NJ, Das R, Emran TB, Sharma R, Chandran D, Islam F, Hossain MJ, Safi SZ, Sweilam SH. 2022. Tacrine derivatives in neurological disorders: focus on molecular mechanisms and neurotherapeutic potential. Oxid Med Cell Longev 2022:1–22. doi:10.1155/2022/7252882 PMC9410840 36035218

[41] Cholo MC, Steel HC, Fourie PB, Germishuizen WA, Anderson R. 2012. Clofazimine: current status and future prospects. J Antimicrob Chemother 67:290–298. doi:10.1093/jac/dkr444 22020137

[42] McGuffin SA, Pottinger PS, Harnisch JP. 2017. Clofazimine in nontuberculous mycobacterial infections: a growing niche. Open Forum Infect Dis 4. doi:10.1093/ofid/ofx147 PMC6124512 30202770

[43] Xu J, Koval A, Katanaev VL. 2023. Clofazimine: a journey of a drug. Biomedicine & Pharmacotherapy 167:115539. doi:10.1016/j.biopha.2023.115539 37742606

[44] Heck AM, Yanovski JA, Calis KA. 2000. Orlistat, a new lipase inhibitor for the management of obesity. Pharmacotherapy 20:270–279. doi:10.1592/phco.20.4.270.34882 10730683 PMC6145169

[45] Katimbwa DA, Oh J, Jang CH, Lim J. 2022. Orlistat, a competitive lipase inhibitor used as an antiobesity remedy, enhances inflammatory reactions in the intestine. Appl Biol Chem 65. doi:10.1186/s13765-022-00712-y

[46] Margolis RL. 2014. Tetrabenazine, depression and suicide: good news. J Huntingtons Dis 3:137–138. doi:10.3233/JHD-140107 25062856

[47] Savva K, Zachariou M, Bourdakou MM, Dietis N, Spyrou GM. 2022. Network-based stage-specific drug repurposing for Alzheimer’s disease. Comput Struct Biotechnol J 20:1427–1438. doi:10.1016/j.csbj.2022.03.013 35386099 PMC8957022

[48] Singu B, Verbeeck RK. 2021. Should codeine still be considered a WHO essential medicine? J Pharm Pharm Sci 24:329–335. doi:10.18433/jpps31639 34192509

[49] Peechakara BV, Tharp JG. 2024. Codeine. StatPearls Publishing. 30252285

[50] Sanford M. 2012. Frovatriptan. CNS Drugs 26:791–811. doi:10.2165/11209380-000000000-00000 22900951

[51] Hurst M, Jarvis B. 2001. Perindopril. Drugs (Abingdon Engl) 61:867–896. doi:10.2165/00003495-200161060-00020 11398915

[52] Alfakih K, Hall AS. 2006. Perindopril. Expert Opin Pharmacother 7:63–71. doi:10.1517/14656566.7.1.63 16370923

[53] Dilger JP, Vidal AM, Liu M, Mettewie C, Suzuki T, Pham A, Demazumder D. 2007. Roles of amino acids and subunits in determining the inhibition of nicotinic acetylcholine receptors by competitive antagonists. Anesthesiology 106:1186–1195. doi:10.1097/01.anes.0000267602.94516.7f 17525594 PMC2367005

[54] Fagerlund MJ, Dabrowski M, Eriksson LI. 2009. Pharmacological characteristics of the inhibition of nondepolarizing neuromuscular blocking agents at human adult muscle nicotinic acetylcholine receptor. Anesthesiology 110:1244–1252. doi:10.1097/ALN.0b013e31819fade3 19417616

[55] Li W, Xie L, Chen Z, Zhu Y, Sun Y, Miao Y, Xu Z, Han X. 2010. Cantharidin, a potent and selective PP2A inhibitor, induces an oxidative stress-independent growth inhibition of pancreatic cancer cells through G2/M cell-cycle arrest and apoptosis. Cancer Sci 101:1226–1233. doi:10.1111/j.1349-7006.2010.01523.x 20331621 PMC11158714

[56] Maher‐Edwards G, De’Ath J, Barnett C, Lavrov A, Lockhart A. 2015. A 24‐week study to evaluate the effect of rilapladib on cognition and cerebrospinal fluid biomarkers of Alzheimer’s disease. A&D Transl Res & Clin Interv 1:131–140. doi:10.1016/j.trci.2015.06.003 PMC5975052 29854933

[57] Corson MA. 2010. Darapladib: an emerging therapy for atherosclerosis. Ther Adv Cardiovasc Dis 4:241–248. doi:10.1177/1753944710375820 20660537

[58] Zhang J, Xu D-L, Liu X-B, Bi S, Zhao T, Sui S-J, Ji X-P, Lu Q-H. 2016. Darapladib, a lipoprotein-associated phospholipase A2 inhibitor, reduces rho kinase activity in atherosclerosis. Yonsei Med J 57:321. doi:10.3349/ymj.2016.57.2.321 26847282 PMC4740522

[59] Zhuo S, Yuan C. 2020. Active site competition is the mechanism for the inhibition of lipoprotein-associated phospholipase A_2_ by detergent micelles or lipoproteins and for the efficacy reduction of darapladib. Sci Rep 10:17232. doi:10.1038/s41598-020-74236-0 33057060 PMC7560626

[60] Sastry BVR, Hemontolor ME, Vidaver PS, Sastry WS, Janson VE. 1999. Influence of halothane on phospholipase A _2_ and enzymatic methylations in the rat retinal membranes. J Ocul Pharmacol Ther 15:165–178. doi:10.1089/jop.1999.15.165 10229494

[61] Ong W-Y, Lu X-R, Ong BK-C, Horrocks LA, Farooqui AA, Lim S-K. 2003. Quinacrine abolishes increases in cytoplasmic phospholipase A2 mRNA levels in the rat hippocampus after kainate-induced neuronal injury. Exp Brain Res 148:521–524. doi:10.1007/s00221-002-1315-2 12582837

[62] Hossain M, Giri P, Kumar GS. 2008. DNA intercalation by quinacrine and methylene blue: a comparative binding and thermodynamic characterization study. DNA Cell Biol 27:81–90. doi:10.1089/dna.2007.0652 17924822

[63] Leite JO, Vaishnav U, Puglisi M, Fraser H, Trias J, Fernandez ML. 2009. A-002 (varespladib), a phospholipase A2 inhibitor, reduces atherosclerosis in guinea pigs. BMC Cardiovasc Disord 9:7. doi:10.1186/1471-2261-9-7 19222850 PMC2653470

[64] Giordanetto F, Pettersen D. 2017. Fragment-based discovery of AZD2716: a novel, potent secreted phospholipase A 2 (sPLA 2) inhibitor for the treatment of coronary artery disease, p 339–348. In DR Samuel Chackalamannil, Ward Simon E (ed), Comprehensive Medicinal Chemistry III. Elsevier. 10.1021/acsmedchemlett.6b00188 PMC5066155 27774123

[65] Oglesby TD, Gorman RR. 1984. The inhibition of arachidonic acid metabolism in human platelets by RHC 80267, a diacylglycerol lipase inhibitor. Biochim Biophys Acta 793:269–277. doi:10.1016/0005-2760(84)90329-1 6424715

[66] Suzuki H, Kito Y, Fukuta H, Yamamoto Y. 2002. Effects of RHC-80267, an inhibitor of diacylglycerol lipase, on excitation of circular smooth muscle of the guinea-pig gastric antrum. J Smooth Muscle Res 38:153–164. doi:10.1540/jsmr.38.153 12713022

[67] Ghisdal P, Vandenberg G, Hamaide M-C, Wibo M, Morel N. 2005. The diacylglycerol lipase inhibitor RHC-80267 potentiates the relaxation to acetylcholine in rat mesenteric artery by anti-cholinesterase action. Eur J Pharmacol 517:97–102. doi:10.1016/j.ejphar.2005.05.036 15958263

[68] Won SJ, Davda D, Labby KJ, Hwang SY, Pricer R, Majmudar JD, Armacost KA, Rodriguez LA, Rodriguez CL, Chong FS, Torossian KA, Palakurthi J, Hur ES, Meagher JL, Brooks CL, Stuckey JA, Martin BR. 2016. Molecular mechanism for isoform-selective inhibition of acyl protein thioesterases 1 and 2 (APT1 and APT2). ACS Chem Biol 11:3374–3382. doi:10.1021/acschembio.6b00720 27748579 PMC5359770

[69] Hernandez JL, Davda D, Cheung See Kit M, Majmudar JD, Won SJ, Gang M, Pasupuleti SC, Choi AI, Bartkowiak CM, Martin BR. 2017. APT2 inhibition restores scribble localization and S -palmitoylation in snail-transformed cells. Cell Chem Biol 24:87–97. doi:10.1016/j.chembiol.2016.12.007 28065656 PMC5362123

[70] Virlogeux A, Scaramuzzino C, Lenoir S, Carpentier R, Louessard M, Genoux A, Lino P, Hinckelmann M-V, Perrier AL, Humbert S, Saudou F. 2021. Increasing brain palmitoylation rescues behavior and neuropathology in Huntington disease mice. Sci Adv 7:eabb0799. doi:10.1126/sciadv.abb0799 33789888 PMC8011966

[71] Dykes SS, Friday E, Pruitt K, Cardelli JA. 2015. The histone deacetylase inhibitor cambinol prevents acidic pHe-induced anterograde lysosome trafficking independently of sirtuin activity. Biochem Biophys Rep 3:83–93. doi:10.1016/j.bbrep.2015.07.015 29124170 PMC5668693

[72] Figuera-Losada M, Stathis M, Dorskind JM, Thomas AG, Bandaru VVR, Yoo S-W, Westwood NJ, Rogers GW, McArthur JC, Haughey NJ, Slusher BS, Rojas C. 2015. Cambinol, a novel inhibitor of neutral sphingomyelinase 2 shows neuroprotective properties. PLoS One 10:e0124481. doi:10.1371/journal.pone.0124481 26010541 PMC4444023

[73] Wawruszak A, Luszczki J, Okon E, Czerwonka A, Stepulak A. 2022. Antagonistic pharmacological interaction between sirtuin inhibitor cambinol and paclitaxel in triple-negative breast cancer cell lines: an isobolographic analysis. Int J Mol Sci 23:6458. doi:10.3390/ijms23126458 35742901 PMC9223454

[74] Luberto C, Hassler DF, Signorelli P, Okamoto Y, Sawai H, Boros E, Hazen-Martin DJ, Obeid LM, Hannun YA, Smith GK. 2002. Inhibition of tumor necrosis factor-induced cell death in MCF7 by a novel inhibitor of neutral sphingomyelinase. J Biol Chem 277:41128–41139. doi:10.1074/jbc.M206747200 12154098

[75] Wan X, Fang Y, Du J, Cai S, Dong H. 2023. GW4869 can inhibit epithelial-mesenchymal transition and extracellular HSP90α in gefitinib-sensitive NSCLC cells. Onco Targets Ther 16:913–922. doi:10.2147/OTT.S428707 38021444 PMC10640835

[76] Dinkins MB, Dasgupta S, Wang G, Zhu G, Bieberich E. 2014. Exosome reduction in vivo is associated with lower amyloid plaque load in the 5XFAD mouse model of Alzheimer’s disease. Neurobiol Aging 35:1792–1800. doi:10.1016/j.neurobiolaging.2014.02.012 24650793 PMC4035236

[77] Kim SY, Moon TC, Chang HW, Son KH, Kang SS, Kim HP. 2002. Effects of tanshinone I isolated from Salvia miltiorrhiza bunge on arachidonic acid metabolism and in vivo inflammatory responses. Phytother Res 16:616–620. doi:10.1002/ptr.941 12410540

[78] Nizamutdinova IT, Lee GW, Lee JS, Cho MK, Son KH, Jeon SJ, Kang SS, Kim YS, Lee JH, Seo HG, Chang KC, Kim HJ. 2008. Tanshinone I suppresses growth and invasion of human breast cancer cells, MDA-MB-231, through regulation of adhesion molecules. Carcinogenesis 29:1885–1892. doi:10.1093/carcin/bgn151 18586687

[79] Huang Y, Yu S-H, Zhen W-X, Cheng T, Wang D, Lin J-B, Wu Y-H, Wang Y-F, Chen Y, Shu L-P, Wang Y, Sun X-J, Zhou Y, Yang F, Hsu C-H, Xu P-F. 2021. Tanshinone I, a new EZH2 inhibitor restricts normal and malignant hematopoiesis through upregulation of MMP9 and ABCG2 Theranostics 11:6891–6904. doi:10.7150/thno.53170 34093860 PMC8171091

[80] Huang X, Jin L, Deng H, Wu D, Shen Q, Quan Z, Zhang C, Guo H-Y. 2022. Research and development of natural product tanshinone I: pharmacology, total synthesis, and structure modifications. Front Pharmacol 13. doi:10.3389/fphar.2022.920411 PMC9315943 35903340

[81] Shiyu S, Zhiyu L, Mao Y, Lin B, Lijia W, Tianbao Z, Jie C, Tingyu L. 2011. Polydatin up-regulates clara cell secretory protein to suppress phospholipase A2 of lung induced by LPS in vivo and in vitro. BMC Cell Biol 12:31. doi:10.1186/1471-2121-12-31 21787397 PMC3199855

[82] Karami A, Fakhri S, Kooshki L, Khan H. 2022. Polydatin: pharmacological mechanisms, therapeutic targets, biological activities, and health benefits. Molecules 27:6474. doi:10.3390/molecules27196474 36235012 PMC9572446

[83] Tang D, Zhang Q, Duan H, Ye X, Liu J, Peng W, Wu C. 2022. Polydatin: a critical promising natural agent for liver protection via antioxidative stress. Oxid Med Cell Longev 2022:9218738. doi:10.1155/2022/9218738 35186191 PMC8853764

[84] Jaeger K-E, Kovacic F. 2014. Determination of lipolytic enzyme activities, p 111–134. Springer New York. 10.1007/978-1-4939-0473-0_12 24818902

[85] Jayachandra K, Gowda MDM, Rudresha GV, Manjuprasanna VN, Urs AP, Nandana MB, Bharatha M, Jameel NM, Vishwanath BS. 2023. Inhibition of sPLA_2_ enzyme activity by cell-permeable antioxidant EUK-8 and downregulation of p38, Akt, and p65 signals induced by sPLA_2_ in inflammatory mouse paw edema model. J Cell Biochem 124:294–307. doi:10.1002/jcb.30366 36585945

[86] Reisner A, Krogfelt KA, Klein BM, Zechner EL, Molin S. 2006. In vitro biofilm formation of commensal and pathogenic Escherichia coli strains: impact of environmental and genetic factors. J Bacteriol 188:3572–3581. doi:10.1128/JB.188.10.3572-3581.2006 16672611 PMC1482849

[87] O’Toole GA. 2011. Microtiter dish biofilm formation assay. J Vis Exp 47:2437. doi:10.3791/2437 PMC3182663 21307833

[88] Leggate J, Allain R, Isaac L, Blais BW. 2006. Microplate fluorescence assay for the quantification of double stranded DNA using SYBR green I dye. Biotechnol Lett 28:1587–1594. doi:10.1007/s10529-006-9128-1 16937249

[89] Alio I, Gudzuhn M, Pérez García P, Danso D, Schoelmerich MC, Mamat U, Schaible UE, Steinmann J, Yero D, Gibert I, Kohl TA, Niemann S, Gröschel MI, Haerdter J, Hackl T, Vollstedt C, Bömeke M, Egelkamp R, Daniel R, Poehlein A, Streit WR. 2020. Phenotypic and transcriptomic analyses of seven clinical Stenotrophomonas maltophilia isolates identify a small set of shared and commonly regulated genes involved in the biofilm lifestyle. Appl Environ Microbiol 86:e02038-20. doi:10.1128/AEM.02038-20 PMC7688217 33097507

[90] Berman HM, Westbrook J, Feng Z, Gilliland G, Bhat TN, Weissig H, Shindyalov IN, Bourne PE. 2000. The protein data bank. Nucleic Acids Res 28:235–242. doi:10.1093/nar/28.1.235 10592235 PMC102472

[91] Sali A, Blundell TL. 1993. Comparative protein modelling by satisfaction of spatial restraints. J Mol Biol 234:779–815. doi:10.1006/jmbi.1993.1626 8254673

[92] Lomize MA, Pogozheva ID, Joo H, Mosberg HI, Lomize AL. 2012. OPM database and PPM web server: resources for positioning of proteins in membranes. Nucleic Acids Res 40:D370–6. doi:10.1093/nar/gkr703 21890895 PMC3245162

[93] OpenEye CMS, Inc. 2022. OEDOCKING 4.3.0.3. Santa Fe, NM. Available from: http://www.eyesopen.com

[94] Software OS. 2019. OMEGA 4.1.1.1. Santa Fe, NM, USA. Available from: http://www.eyesopen.com

[95] Schauperl M, Nerenberg PS, Jang H, Wang L-P, Bayly CI, Mobley DL, Gilson MK. 2020. Non-bonded force field model with advanced restrained electrostatic potential charges (RESP2). Commun Chem 3. doi:10.1038/s42004-020-0291-4 PMC8204736 34136662

[96] Frisch MJ, Trucks GW, Schlegel HB, Scuseria GE, Robb MA, Cheeseman JR, Scalmani G, Barone V, Petersson GA, Nakatsuji H, Li X, Caricato M, et al.. 2016. Gaussian 16 Rev. A.03. Wallingford, CT.

[97] Morris GM, Goodsell DS, Halliday RS, Huey R, Hart WE, Belew RK, Olson AJ. 1998. Automated docking using a Lamarckian genetic algorithm and an empirical binding free energy function. J Comput Chem 19:1639–1662. doi:10.1002/(SICI)1096-987X(19981115)19:14<1639::AID-JCC10>3.0.CO;2-B

[98] Dittrich J, Schmidt D, Pfleger C, Gohlke H. 2019. Converging a knowledge-based scoring function: drugScore^2018^. J Chem Inf Model 59:509–521. doi:10.1021/acs.jcim.8b00582 30513206

[99] Murzyn K, Róg T, Pasenkiewicz-Gierula M. 2005. Phosphatidylethanolamine-phosphatidylglycerol bilayer as a model of the inner bacterial membrane. Biophys J 88:1091–1103. doi:10.1529/biophysj.104.048835 15556990 PMC1305115

[100] Martínez L, Andrade R, Birgin EG, Martínez JM. 2009. PACKMOL: a package for building initial configurations for molecular dynamics simulations. J Comput Chem 30:2157–2164. doi:10.1002/jcc.21224 19229944

[101] Schott-Verdugo S, Gohlke H. 2019. PACKMOL-memgen: a simple-to-use, generalized workflow for membrane-protein-lipid-bilayer system building. J Chem Inf Model 59:2522–2528. doi:10.1021/acs.jcim.9b00269 31120747

[102] Case DA, Aktulga HM, Belfon K, Cerutti DS, Cisneros GA, Cruzeiro VWD, Forouzesh N, Giese TJ, Götz AW, Gohlke H, et al.. 2023. AmberTools. J Chem Inf Model 63:6183–6191. doi:10.1021/acs.jcim.3c01153 37805934 PMC10598796

[103] Maier JA, Martinez C, Kasavajhala K, Wickstrom L, Hauser KE, Simmerling C. 2015. ff14SB: improving the accuracy of protein side chain and backbone parameters from ff99SB. J Chem Theory Comput 11:3696–3713. doi:10.1021/acs.jctc.5b00255 26574453 PMC4821407

[104] Dickson CJ, Walker RC, Gould IR. 2022. Lipid21: complex lipid membrane simulations with AMBER. J Chem Theory Comput 18:1726–1736. doi:10.1021/acs.jctc.1c01217 35113553 PMC9007451

[105] He X, Man VH, Yang W, Lee T-S, Wang J. 2020. A fast and high-quality charge model for the next generation general AMBER force field. J Chem Phys 153:114502. doi:10.1063/5.0019056 32962378 PMC7728379

[106] Zhao C-L, Zhao D-X, Bei C-C, Meng X-N, Li S, Yang Z-Z. 2019. Seven-site effective pair potential for simulating liquid water. J Phys Chem B 123:4594–4603. doi:10.1021/acs.jpcb.9b03149 31063377

[107] Li P, Song LF, Merz KM. 2015. Systematic parameterization of monovalent ions employing the nonbonded model. J Chem Theory Comput 11:1645–1657. doi:10.1021/ct500918t 26574374

[108] Sengupta A, Li Z, Song LF, Li P, Merz KM. 2021. Parameterization of monovalent ions for the OPC3, OPC, TIP3P-FB, and TIP4P-FB water models. J Chem Inf Model 61:869–880. doi:10.1021/acs.jcim.0c01390 33538599 PMC8173365

[109] Kräutler V, van Gunsteren WF, Hünenberger PH. 2001. A fast SHAKE algorithm to solve distance constraint equations for small molecules in molecular dynamics simulations. J Comput Chem 22:501–508. doi:10.1002/1096-987X(20010415)22:5<501::AID-JCC1021>3.0.CO;2-V

[110] Le Grand S, Götz AW, Walker RC. 2013. SPFP: speed without compromise—a mixed precision model for GPU accelerated molecular dynamics simulations. Comput Phys Commun 184:374–380. doi:10.1016/j.cpc.2012.09.022

[111] Quigley D, Probert MIJ. 2004. Langevin dynamics in constant pressure extended systems. J Chem Phys 120:11432–11441. doi:10.1063/1.1755657 15268177

[112] Berendsen HJC, Postma JPM, van Gunsteren WF, DiNola A, Haak JR. 1984. Molecular dynamics with coupling to an external bath. J Chem Phys 81:3684–3690. doi:10.1063/1.448118

[113] Lin Y, Pan D, Li J, Zhang L, Shao X. 2017. Application of berendsen barostat in dissipative particle dynamics for nonequilibrium dynamic simulation. J Chem Phys 146:124108. doi:10.1063/1.4978807 28388109

[114] Roe DR, Cheatham TE. 2013. PTRAJ and CPPTRAJ: software for processing and analysis of molecular dynamics trajectory data. J Chem Theory Comput 9:3084–3095. doi:10.1021/ct400341p 26583988

[115] Miller BR, McGee TD, Swails JM, Homeyer N, Gohlke H, Roitberg AE. 2012. MMPBSA.py: an efficient program for end-state free energy calculations. J Chem Theory Comput 8:3314–3321. doi:10.1021/ct300418h 26605738

[116] Greene DA, Qi R, Nguyen R, Qiu T, Luo R. 2019. Heterogeneous dielectric implicit membrane model for the calculation of MMPBSA binding free energies. J Chem Inf Model 59:3041–3056. doi:10.1021/acs.jcim.9b00363 31145610 PMC7197397

[117] Gohlke H, Case DA. 2004. Converging free energy estimates: MM-PB(GB)SA studies on the protein-protein complex Ras-Raf. J Comput Chem 25:238–250. doi:10.1002/jcc.10379 14648622

[118] Gohlke H, Kiel C, Case DA. 2003. Insights into protein-protein binding by binding free energy calculation and free energy decomposition for the Ras-Raf and Ras-RalGDS complexes. J Mol Biol 330:891–913. doi:10.1016/s0022-2836(03)00610-7 12850155

[119] Frieg B, Gremer L, Heise H, Willbold D, Gohlke H. 2020. Binding modes of thioflavin T and congo red to the fibril structure of amyloid-β(1-42). Chem Commun (Camb) 56:7589–7592. doi:10.1039/d0cc01161d 32510059

[120] McQuarrie DA. 1976. Statistical mechanics. Harper & Row, New York.

[121] Janin J. 1996. Quantifying biological specificity: the statistical mechanics of molecular recognition. Proteins 25:438–445. doi:10.1002/prot.4 8865339

[122] Gilson MK, Given JA, Bush BL, McCammon JA. 1997. The statistical-thermodynamic basis for computation of binding affinities: a critical review. Biophys J 72:1047–1069. doi:10.1016/S0006-3495(97)78756-3 9138555 PMC1184492

[123] Luo H, Sharp K. 2002. On the calculation of absolute macromolecular binding free energies. Proc Natl Acad Sci USA 99:10399–10404. doi:10.1073/pnas.162365999 12149474 PMC124926

[124] Vollan HS, Tannæs T, Yamaoka Y, Bukholm G. 2012. In silico evolutionary analysis of Helicobacter pylori outer membrane phospholipase A (OMPLA). BMC Microbiol 12:206. doi:10.1186/1471-2180-12-206 22974200 PMC3490997

[125] Bender J, Rydzewski K, Broich M, Schunder E, Heuner K, Flieger A. 2009. Phospholipase PlaB of Legionella pneumophila represents a novel lipase family. Journal of Biological Chemistry 284:27185–27194. doi:10.1074/jbc.M109.026021 19640837 PMC2785646

[126] Wishart DS. 2006. DrugBank: a comprehensive resource for in silico drug discovery and exploration. Nucleic Acids Res 34:D668–D672. doi:10.1093/nar/gkj067 16381955 PMC1347430

[127] Wishart DS, Knox C, Guo AC, Cheng D, Shrivastava S, Tzur D, Gautam B, Hassanali M. 2008. DrugBank: a knowledgebase for drugs, drug actions and drug targets. Nucleic Acids Res 36:D901–D906. doi:10.1093/nar/gkm958 18048412 PMC2238889

[128] Thi MTT, Wibowo D, Rehm BHA. 2020. Pseudomonas aeruginosa biofilms. IJMS 21:8671. doi:10.3390/ijms21228671 33212950 PMC7698413

[129] Tuon FF, Dantas LR, Suss PH, Tasca Ribeiro VS. 2022. Pathogenesis of the Pseudomonas aeruginosa biofilm: a review. Pathogens 11:300. doi:10.3390/pathogens11030300 35335624 PMC8950561

[130] Soares A, Caron F, Etienne M. 2019. Commentary: tolerance and resistance of Pseudomonas aeruginosa biofilms to antimicrobial agents-how P. aeruginosa can escape antibiotics. Front Microbiol 10. doi:10.3389/fmicb.2019.02164 PMC6759825 31620114

[131] Yin R, Cheng J, Wang J, Li P, Lin J. 2022. Treatment of Pseudomonas aeruginosa infectious biofilms: challenges and strategies. Front Microbiol 13. doi:10.3389/fmicb.2022.955286 PMC9459144 36090087

[132] Dai J, Luo W, Hu F, Li S. 2024. In vitro inhibition of Pseudomonas aeruginosa PAO1 biofilm formation by DZ2002 through regulation of extracellular DNA and alginate production. Front Cell Infect Microbiol 13. doi:10.3389/fcimb.2023.1333773 PMC10806038 38268790

[133] Yayan J, Ghebremedhin B, Rasche K. 2015. Antibiotic resistance of Pseudomonas aeruginosa in pneumonia at a single University hospital center in Germany over a 10-year period. PLoS One 10:e0139836. doi:10.1371/journal.pone.0139836 26430738 PMC4592231

[134] Odds FC. 2003. Synergy, antagonism, and what the chequerboard puts between them. J Antimicrob Chemother 52:1–1. doi:10.1093/jac/dkg301 12805255

[135] Tangy F, Moukkadem M, Vindimian E, Capmau ML, Le Goffic F. 1985. Mechanism of action of gentamicin components. Eur J Biochem 147:381–386. doi:10.1111/j.1432-1033.1985.tb08761.x 3882427

[136] Falagas ME, Kasiakou SK, Saravolatz LD. 2005. Colistin: the revival of polymyxins for the management of multidrug-resistant gram-negative bacterial infections. Clin Infect Dis 40:1333–1341. doi:10.1086/429323 15825037

[137] Lipman B, Neu HC. 1988. Imipenem: a new carbapenem antibiotic. Med Clin North Am 72:567–579. doi:10.1016/s0025-7125(16)30759-3 3280907

[138] Perry CM, Markham A. 1999. Piperacillin/tazobactam. Drugs (Abingdon Engl) 57:805–843. doi:10.2165/00003495-199957050-00017 10353303

[139] Liao C, Huang X, Wang Q, Yao D, Lu W. 2022. Virulence factors of Pseudomonas aeruginosa and antivirulence strategies to combat its drug resistance. Front Cell Infect Microbiol 12. doi:10.3389/fcimb.2022.926758 PMC9299443 35873152

[140] Mishra AS, Vasanthan M, Malliappan SP. 2024. Drug repurposing: a leading strategy for new threats and targets. ACS Pharmacol Transl Sci 7:915–932. doi:10.1021/acsptsci.3c00361 38633585 PMC11019736

[141] Hassan A, Blanchard N. 2022. Microbial (co)infections: powerful immune influencers. PLoS Pathog 18:e1010212. doi:10.1371/journal.ppat.1010212 35113966 PMC8812865

[142] Karagüzel A, Koçak Aslan E, Gündüz MG. 2025. From sulfa drugs to new antibacterial agents: advances in chemical modification of approved sulfonamides. Drug Dev Res 86:e70191. doi:10.1002/ddr.70191 41259286

[143] Larsen EM, Johnson RJ. 2019. Microbial esterases and ester prodrugs: an unlikely marriage for combating antibiotic resistance. Drug Dev Res 80:33–47. doi:10.1002/ddr.21468 30302779 PMC6377847

[144] Essandoh K, Yang L, Wang X, Huang W, Qin D, Hao J, Wang Y, Zingarelli B, Peng T, Fan G-C. 2015. Blockade of exosome generation with GW4869 dampens the sepsis-induced inflammation and cardiac dysfunction. Biochimica et Biophysica Acta (BBA) - Molecular Basis of Disease 1852:2362–2371. doi:10.1016/j.bbadis.2015.08.010 26300484 PMC4581992

[145] Jung AL, Herkt CE, Schulz C, Bolte K, Seidel K, Scheller N, Sittka-Stark A, Bertrams W, Schmeck B. 2017. Legionella pneumophila infection activates bystander cells differentially by bacterial and host cell vesicles. Sci Rep 7:6301. doi:10.1038/s41598-017-06443-1 28740179 PMC5524687

[146] Lister PD, Wolter DJ, Hanson ND. 2009. Antibacterial-resistant Pseudomonas aeruginosa: clinical impact and complex regulation of chromosomally encoded resistance mechanisms. Clin Microbiol Rev 22:582–610. doi:10.1128/CMR.00040-09 19822890 PMC2772362

[147] Tabatadze N, Savonenko A, Song H, Bandaru VVR, Chu M, Haughey NJ. 2010. Inhibition of neutral sphingomyelinase-2 perturbs brain sphingolipid balance and spatial memory in mice. J Neurosci Res 88:2940–2951. doi:10.1002/jnr.22438 20629193 PMC2919585

[148] Peng Y, Zhao M, Hu Y, Guo H, Zhang Y, Huang Y, Zhao L, Chai Y, Wang Z. 2022. Blockade of exosome generation by GW4869 inhibits the education of M2 macrophages in prostate cancer. BMC Immunol 23. doi:10.1186/s12865-022-00514-3 PMC9361607 35941539

[149] Grassmé H, Becker KA. 2013. Bacterial infections and ceramide, p 305–320. In Gulbins E, Petrache I (ed), Sphingolipids in disease. Springer Vienna.

[150] Duarte C, Akkaoui J, Yamada C, Ho A, Mao C, Movila A. 2020. Elusive roles of the different ceramidases in human health, pathophysiology, and tissue regeneration. Cells 9:1379. doi:10.3390/cells9061379 32498325 PMC7349419

