# Supplementary material for: A novel approach to combat Pseudomonas aeruginosa: repurposing pharmaceuticals for inhibition of phospholipase A
Source: Microbiol Spectr. 2026 Jan 21;14(3):e01304-25. doi: 10.1128/spectrum.01304-25 (PMC12955470; doi:10.1128/spectrum.01304-25)
Supplement: Supplemental material — Tables S1 to S3; Fig. S1 to S9. [file spectrum.01304-25-s0001.docx]

**Supplementary information**

**A novel approach to combat *Pseudomonas aeruginosa*: repurposing pharmaceuticals for inhibition of phospholipase A**

Matea Modric^1^, Rocco Gentile^2^, Raphael Moll^3^, Ifey Alio^3^, Wolfgang R. Streit^3^, Karl-Erich Jaeger^1^, Holger Gohlke^2,4,*^ and Filip Kovacic^1,5,6*^

^1^Institute of Molecular Enzyme Technology, Heinrich Heine University Düsseldorf, Forschungszentrum Jülich GmbH, D-52425 Jülich, Germany

^2^Institute for Pharmaceutical and Medicinal Chemistry, Heinrich Heine University Düsseldorf, 40225 Düsseldorf, Germany

^3^Department of Microbiology and Biotechnology, University of Hamburg, Ohnhorststr. 18, 22609 Hamburg, Germany

^4^Institute of Bio- and Geosciences (IBG-4: Bioinformatics), Forschungszentrum Jülich GmbH, 52425 Jülich, Germany

^5^ Department of Surgery, Massachusetts General Hospital, Boston, Massachusetts, USA

^6^ Department of Microbiology, Harvard Medical School, Boston, Massachusetts, USA

* Corresponding authors: [fkovacic@mgh.harvard.edu](mailto:fkovacic@mgh.harvard.edu), [gohlke@uni-duesseldorf.de](mailto:gohlke@uni-duesseldorf.de)

| **Compound** | **Company** | **Article number** | ***c* (stock) / mM** | **Solvent** |
| --- | --- | --- | --- | --- |
| GW4869 | Selleckchem | S7609 | 1.73 | DMSO |
| Codeine | Sigma-Aldrich | C-006 | 3.34 | MeOH |
| Cambinol | Selleckchem | S5913 | 10 | DMSO |
| Darapladib | Selleckchem | S7520 | 10 | DMSO |
| Rilapladib | AdooQ Bioscience | A13229 | 10 | DMSO |
| Varespladib | Selleckchem | S1110 | 10 | DMSO |
| RHC80267 | Selleckchem | S0766 | 10 | DMSO |
| ML348 | Selleckchem | S6564 | 10 | DMSO |
| Vecuronium bromide | Sigma-Aldrich | S1405 | 10 | H_2_O |
| Clofazimine | Sigma-Aldrich | C8895 | 10 | DMSO |
| Frovatriptan succinate monohydrate | Sigma-Aldrich | SML1291 | 20 | H_2_O |
| Perindopril erbumine | Sigma-Aldrich | S1506 | 20 | H_2_O |
| Tetrabenazine | Sigma-Aldrich | T2952 | 30 | DMSO |
| Pancuronium dibromide | Sigma-Aldrich | S2497 | 30 | H_2_O |
| Galantamine hydrobromide | Merck | PHR1623 | 30 | H_2_O |
| Donepezil hydrochloride | Merck | PHR1584 | 30 | H_2_O |
| Rivastigmine tartate | Merck | PHR1867 | 30 | H_2_O |
| Tacrine hydrochloride | Merck | A79922 | 30 | H_2_O |
| Orlistat | Sigma-Aldrich | O4139 | 30 | DMSO |
| Tanshinone I | Selleckchem | S2364 | 30 | DMSO |
| Quanicrine hydrochloride | Selleckchem | S4255 | 50 | H_2_O |
| Cantharidin | Sigma-Aldrich | C7632 | 100 | DMSO |
| Polydatin | Selleckchem | S2390 | 100 | DMSO |

**Table S1.** **Preparation of pharmaceuticals.**

**Table S2. Settings of confocal laser scanning microscope 800 for the visualization of biofilm.**

| **Settings** | **Propidium iodide (PI)** | **SYTO9** |
| --- | --- | --- |
| Objective | C-Apochromat 63W/1.20W Corr; UV VIS IR | C-Apochromat 63W/1.20W Corr; UV VIS IR |
| Scan mode | frame | frame |
| Frame Size | 1024 x 1024 | 1024 x 1024 |
| Bits per pixel | 16 | 16 |
| Scan direction | bidirectional | bidirectional |
| Scan speed | 6 | 6 |
| Laser wavelength | 561 nm | 488 nm |
| Laser intensity | variable | variable |
| Pinhole | 51 µm | 51 µm |
| Master Gain | 700 V | 650 V |
| Digital offset | 0 | 0 |
| Digital gain | 1.0 | 1.0 |

**Table S3. Structure and sequence identity and similarity towards PlaF.**

| **Target^a^** | **Binding site identity wrt. PlaF [%]** | **Binding site similarity wrt. PlaF [%]** | **Residues correctly aligned with PyMOL [%]** | **RMSD wrt. PlaF after omitting not aligned residues [Å]** | **Binding site residues with RMSD < 1.5 Å [%]** | **Binding site residues not aligned [%]** |
| --- | --- | --- | --- | --- | --- | --- |
| O60906 | 9.6 | 25.0 | 23.9 | 17.3 | 0.0 | 60.8 |
| Q9BV23 | 32.7 | 59.6 | 67.1 | 1.1 | 19.2 | 49.0 |
| Q9H6B9 | 19.2 | 34.6 | 50.0 | 2.2 | 0.0 | 54.9 |
| Q8IUS5 | 13.5 | 32.7 | 43.4 | 1.1 | 0.0 | 58.8 |

^[a]^ The same color code is used in Figures S5 and S8.


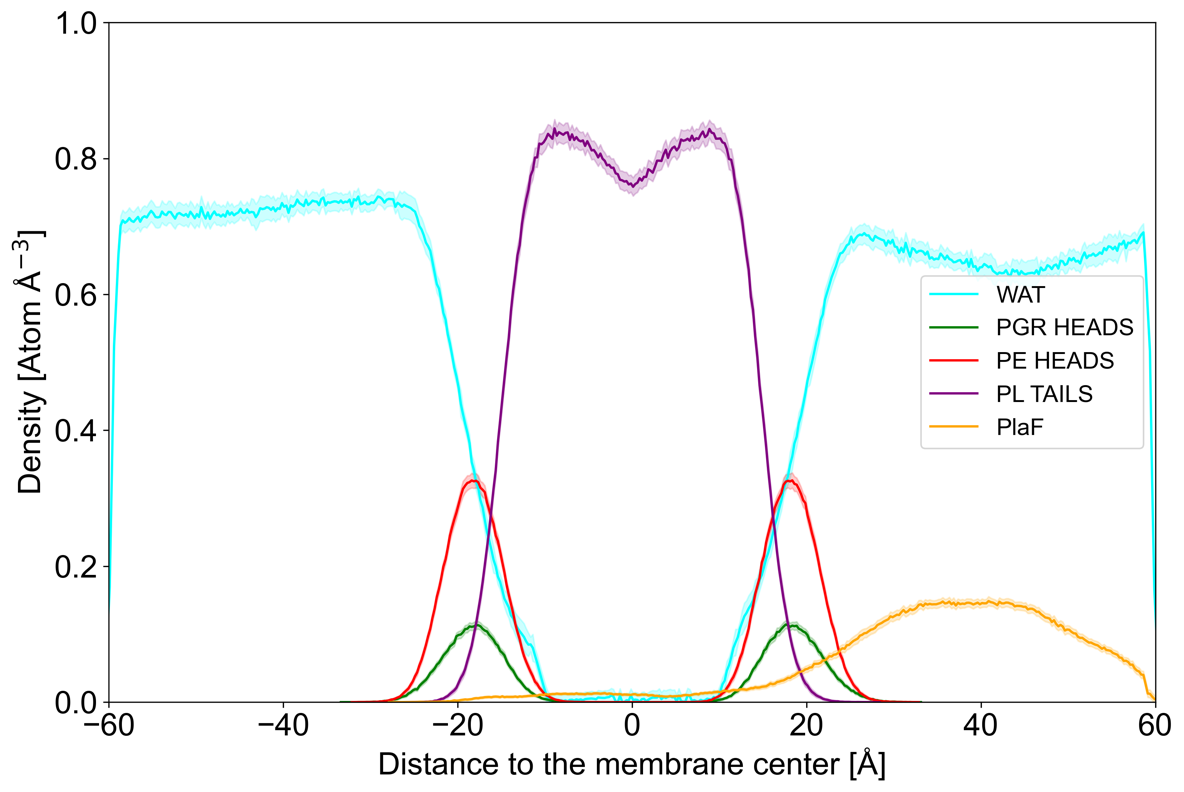


**Figure S1**. Atom density profiles of membrane components averaged over 5 independent unbiased MD simulations of t-PlaF configuration bound to GW4869 for the phospholipid (PL) tails and head groups (PE and PGR). The obtained shapes correspond with those generally found by experiments and MD simulations of PlaF (1).
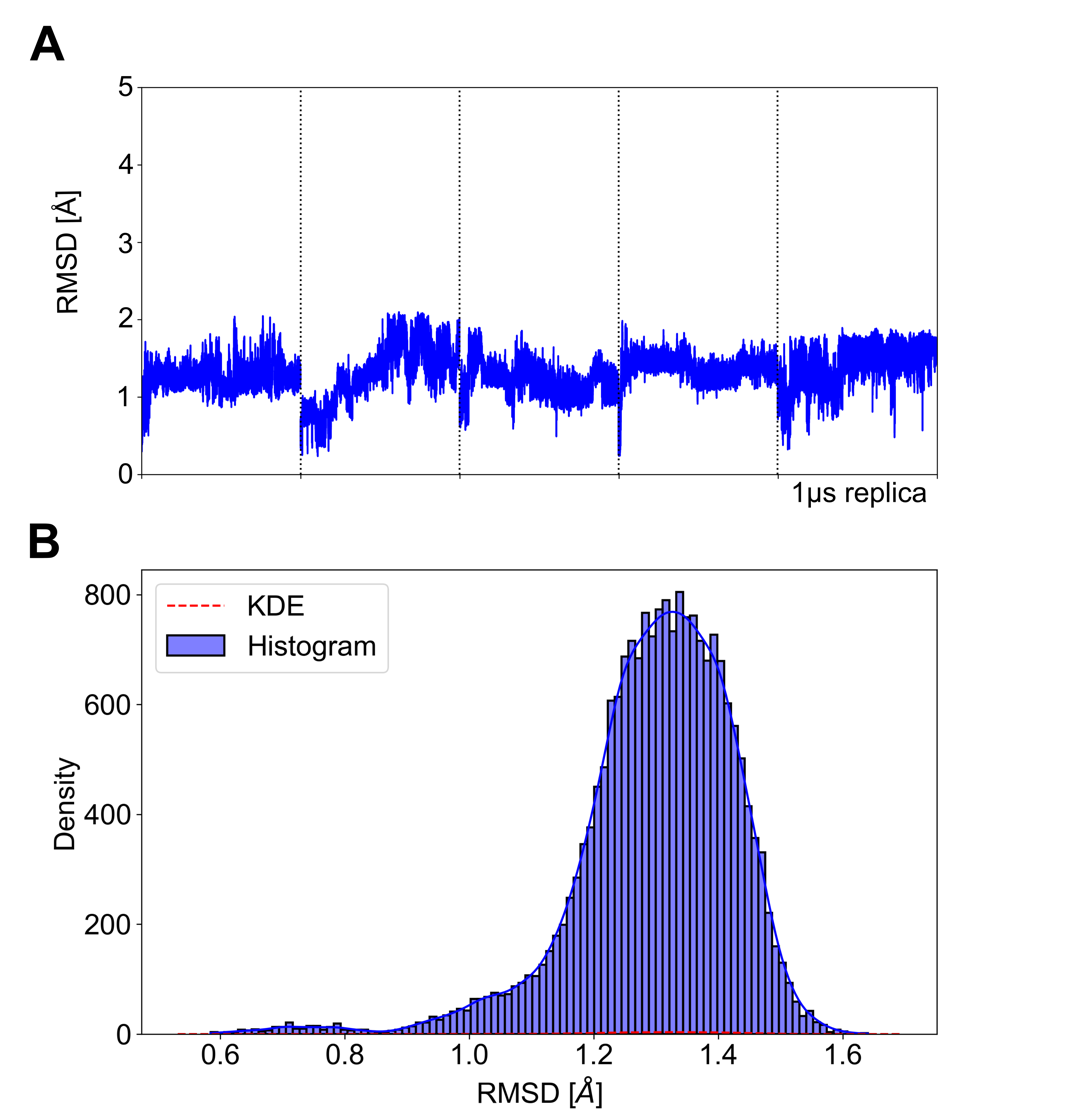


**Figure S2**. Structural mobility of (A) GW4869 molecule in MD simulations and (B) frequency distribution of the calculated RMSD of panel (A). In all replicas, the GW4869 molecules preserve their position after 1 μs with respect to the first frame (RMSD < 2 Å), revealing stably bound poses. The RMSD of all ligand atoms was computed after superimposing the C_α_ atoms of t-PlaF. Dotted lines separate independent replicas. (B) The histograms distribution confirms the very low RMSD value across the different replicas. The kernel density (KDE) is also depicted as dotted red lines.


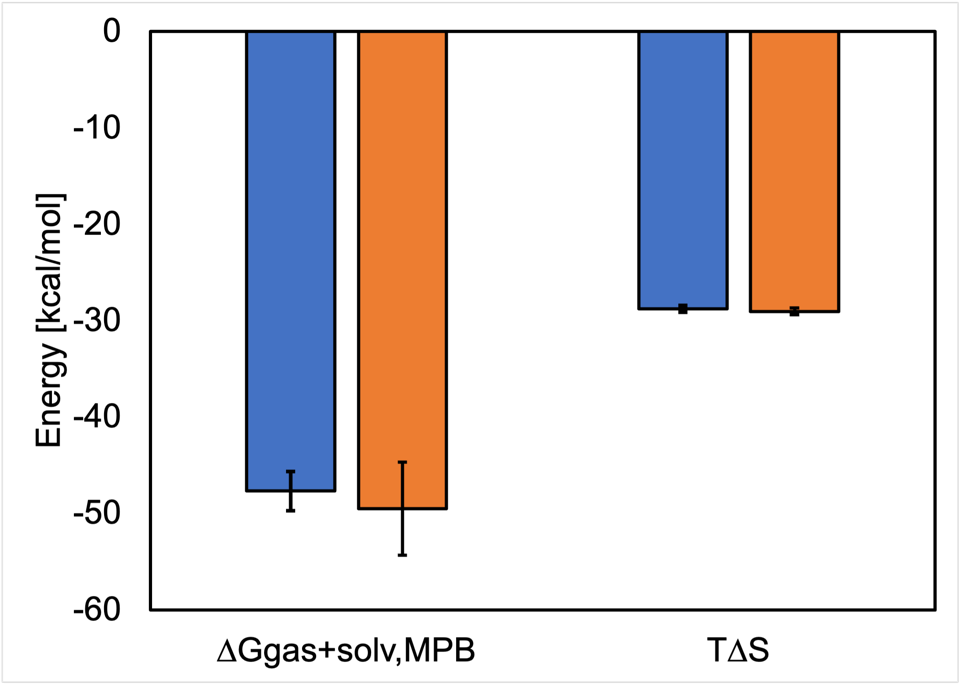


**Figure S3**. MM-PBSA binding effective energy computations and NMA entropy computations of GW4869 are converged. Blue bars describe the calculated energy for the first half of the replica. Orange histograms describe the results for the second half of the replica. The error bars represent the calculated SEM.


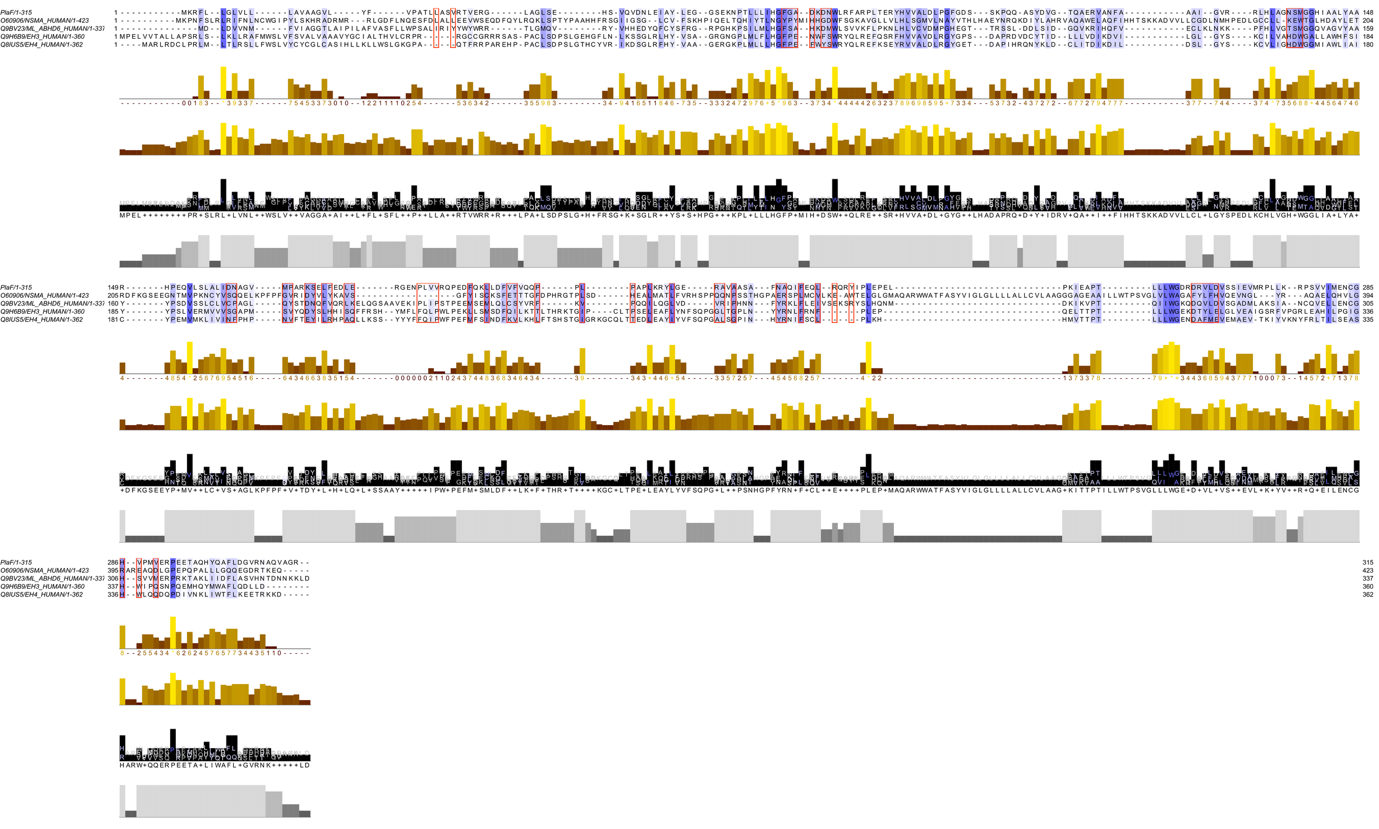


**Figure S4**. Sequence similarity comparison between *P. aeruginosa* PlaF (first row), human sphingomyelinase nSMase, and the closest human PLAs. A basic local sequence search with BLAST (2) was performed to identify similar human sequences: besides human sphingomyelinase nSMase2 (for which GW4869 is a known inhibitor, Uniprot_ID: O60906), the human monoacylglycerol lipase ABHD6 (Q9BV23) and the epoxide hydrolases 3 (Q9H6B9) and 4 (Q8IUS5) were identified. The degree of conservation is shown in a blue scale, with higher intensity indicating higher similarity. The binding site residues identified for GW4869 in PlaF in this study are indicated with a red frame.


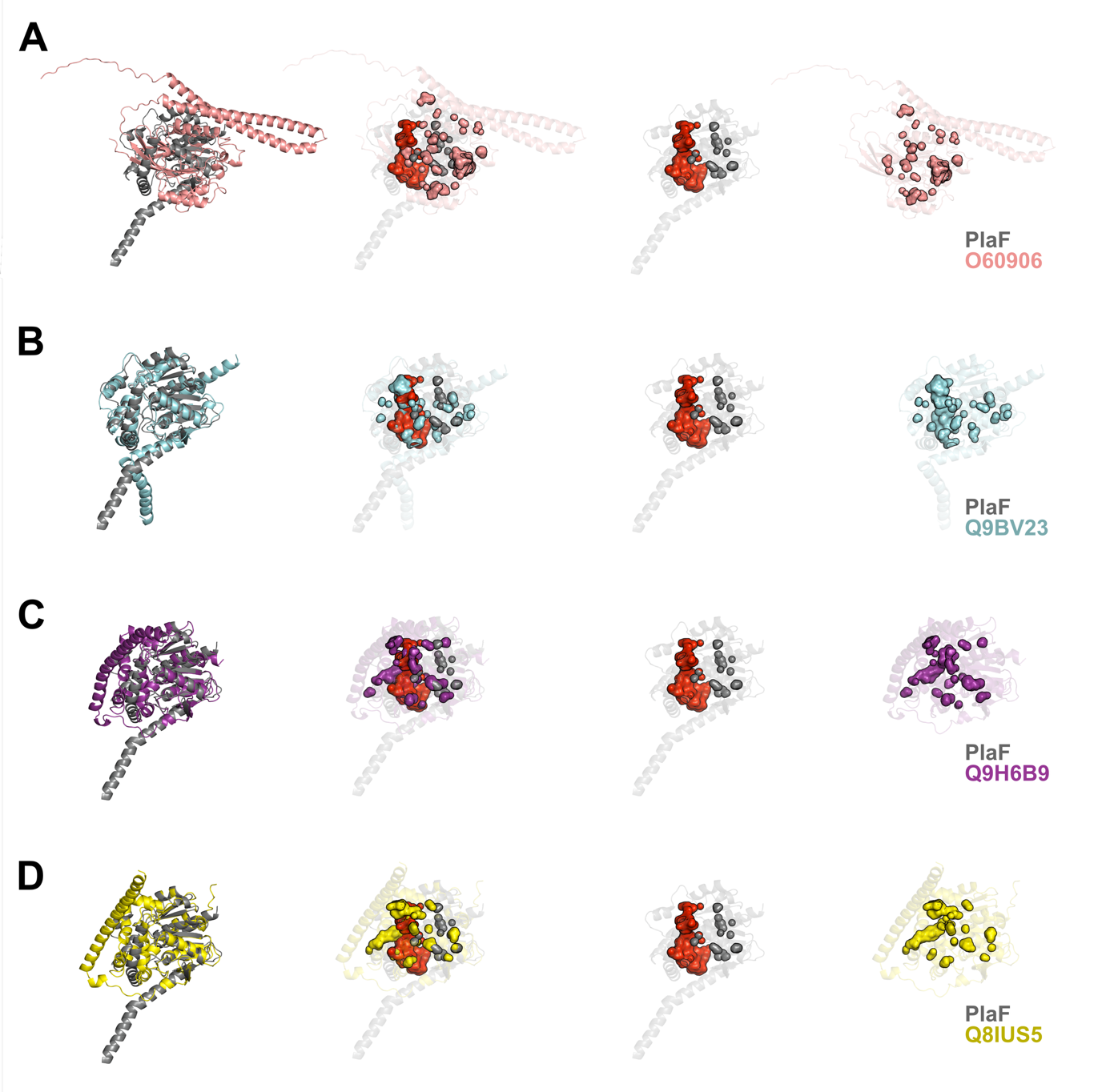


**Figure S5**. Structural superimposition of PlaF (grey) with the sphingomyelinase nSMase (Uniprot_ID: O60906) (salmon) (A), the monoacylglycerol lipase ABHD6 (Uniprot_ID: Q9BV23) (cyan) (B), the epoxide hydrolase 3 (Uniprot_ID: Q9H6B9) (magenta) (C), and the epoxide hydrolase 4 (Uniprot_ID: Q8IUS5) (yellow) (D). Cavities within three solvent radii are shown as surfaces. The GW4869 cavity in PlaF is colored in red, the cavities in the other targets are colored according to the cartoon representation. The binding site cavity slightly overlaps only with ABHD6 (B) but does not do so in the other structures, suggesting high structural diversity*.*


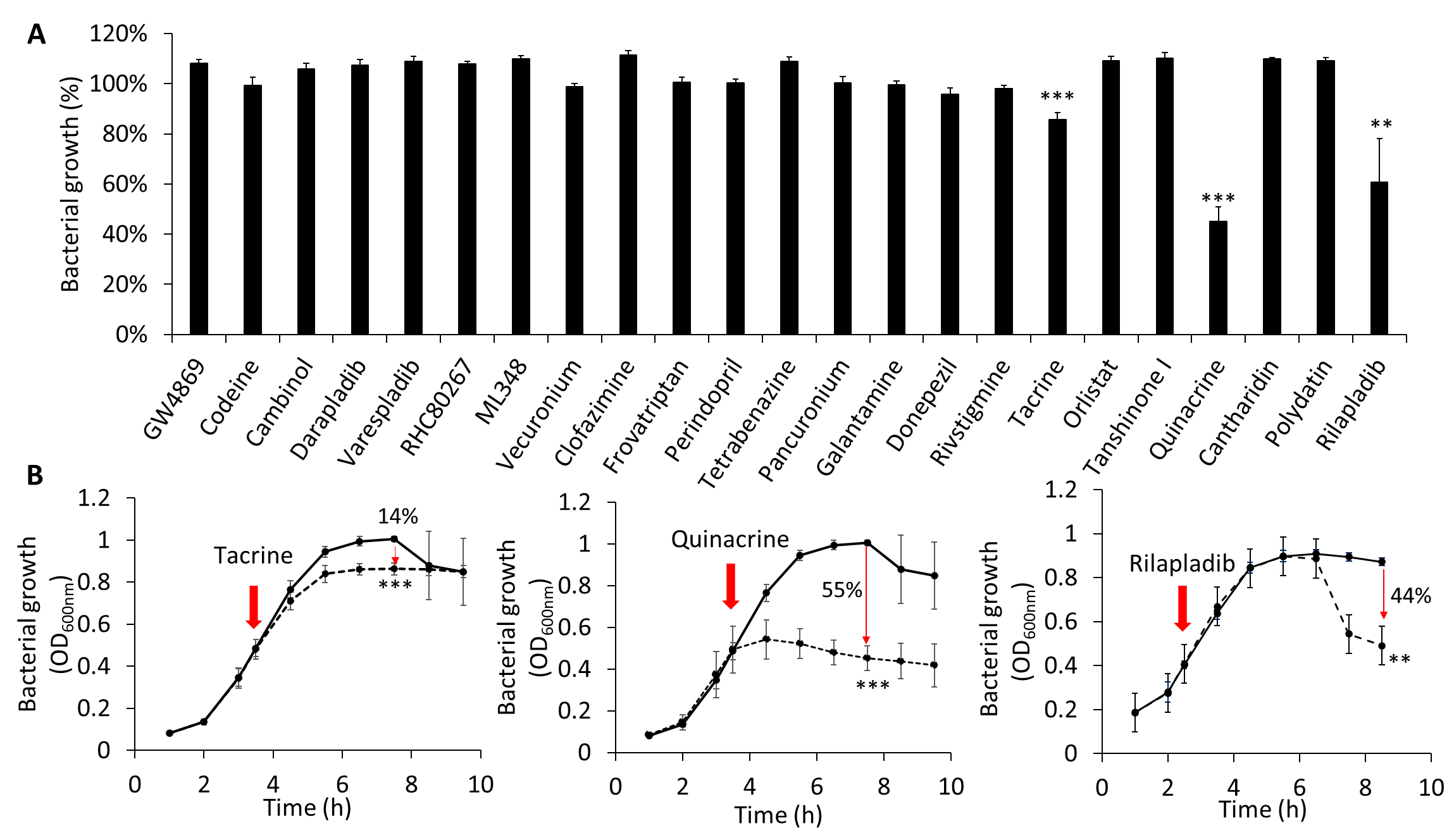


**Figure S6:** **Potential PLA inhibitors impact the growth of *E. coli* ATCC 25922*.*** **A)** Effect of 23 pharmaceuticals on the planktonic growth of *E. coli* after 5 h incubation with compounds. Relative growth values represent optical densities (OD_600nm_) of compound-treated cultures compared to the OD_600nm_ of respective solvent-treated cultures set to 100 %. Results are shown as the mean ± S.D. of 6 biological replicates (n = 6) from two independent experiments. *t*-test of normally distributed values, ***p* < 0.01, ****p* < 0.001. **B)** Growth curves of *E. coli* treated with tacrine (500 µM), quinacrine (300 µM), and rilapladib (100 µM). Bacterial cultures were grown in LB medium at 37 °C with shaking at 1000 rpm in plastic MTP. Red arrows indicate beginning of treatment. Results are the mean ± S.D. of three independent experiments (n = 3). *t*-test of normally distributed values, ***p* < 0.01, ****p* < 0.001.

**
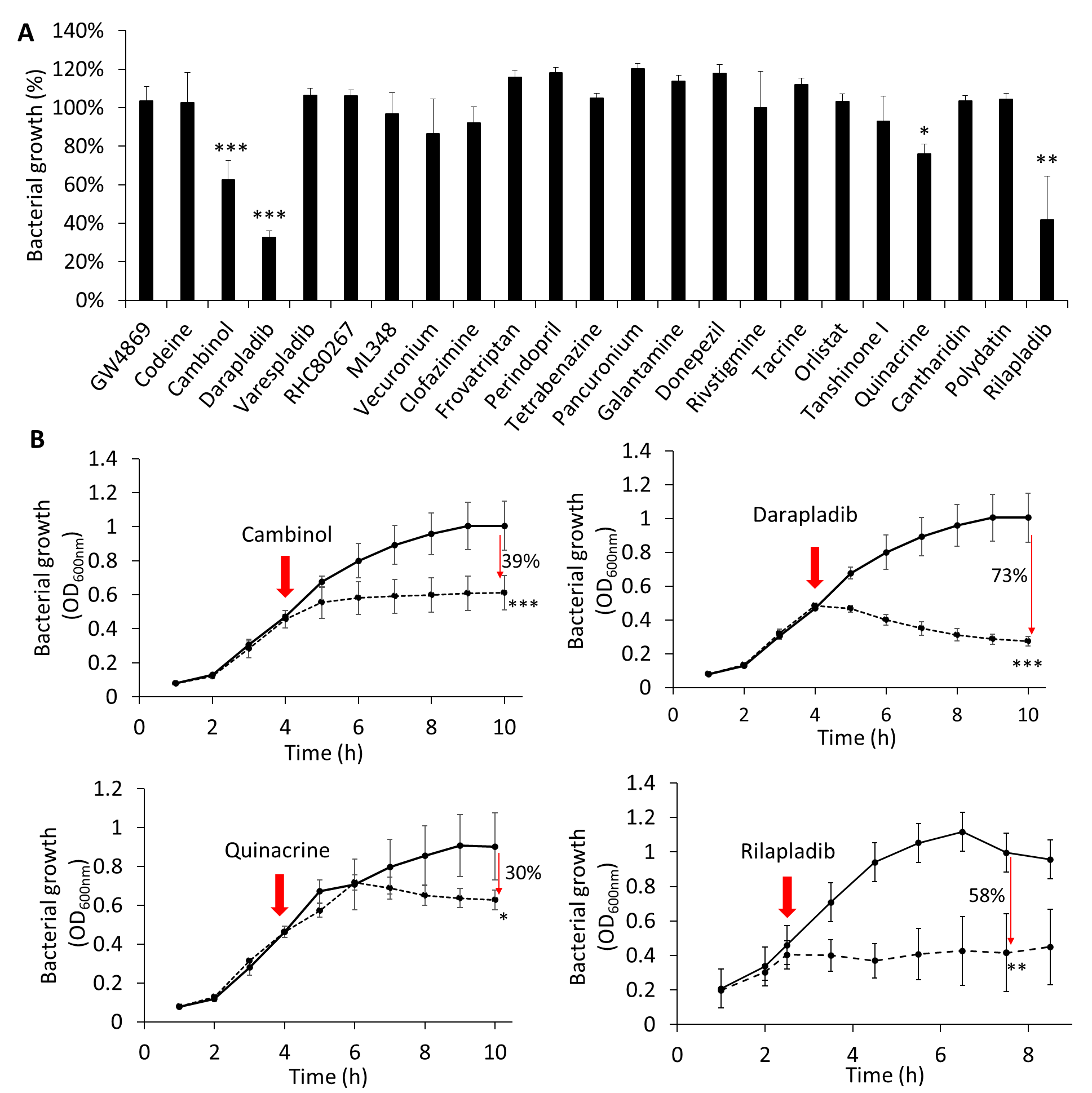
Figure S7:** **Potential PLA inhibitors impact the growth of *S. aureus*** ATCC 25923***.*** **A)** Effect of 23 pharmaceuticals on the planktonic growth of *S. aureus* after 5 h incubation with compounds. Relative growth values represent optical densities (OD_600nm_) of compound-treated cultures compared to the OD_600nm_ of respective solvent-treated cultures set to 100 %. Results are shown as the mean ± S.D. of 6 biological replicates (n = 6) from two independent experiments. *t*-test of normally distributed values, **p* < 0.05, ***p* < 0.01, ****p* < 0.001. **B)** Growth curves of *E. coli* treated with tacrine (500 µM), quinacrine (300 µM), and rilapladib (100 µM). Bacterial cultures were grown in LB medium at 37 °C with shaking at 1000 rpm in plastic MTP. Red arrows indicate beginning of treatment. Results are the mean ± S.D. of three independent experiments (n = 3). *t*-test of normally distributed values, ***p* < 0.01, ****p* < 0.001.

**
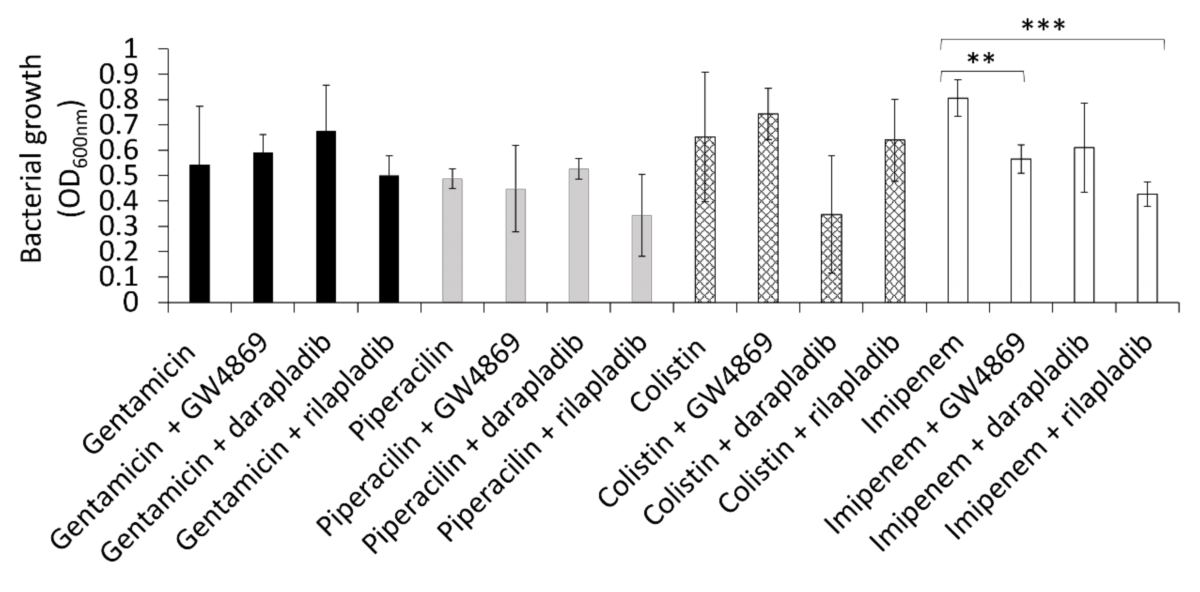
**

**Figure S8.** Combination treatment of four last resort antibiotics against *P. aeruginosa* with GW4869, darapladib, and rilapladib. Gentamicin (0.5 mg/L = 0.70 µM), piperacillin (2 mg/L = 3.71 µM), colistin (1 mg/L = 0.71 µM), and imipenem (2 mg/L = 6.30 µM) were incubated with GW4869 (17.3 µM), darapladib (100 µM), and rilapladib (100 µM) for 5 h at 37 °C with shaking at 1000 rpm. GW4869 and rilapladib together with imipenem showed significantly reduced growth of *P. aeruginosa* PA01 compared to the samples treated with imipenem only. Other combinations of antibiotics and drugs did not significantly reduce growth compared to samples treated with only the antibiotics. Results are shown as the mean of four biological replicates ± S.D. *t*-test for normally distributed values, ***p* < 0.01, ****p* < 0.001.


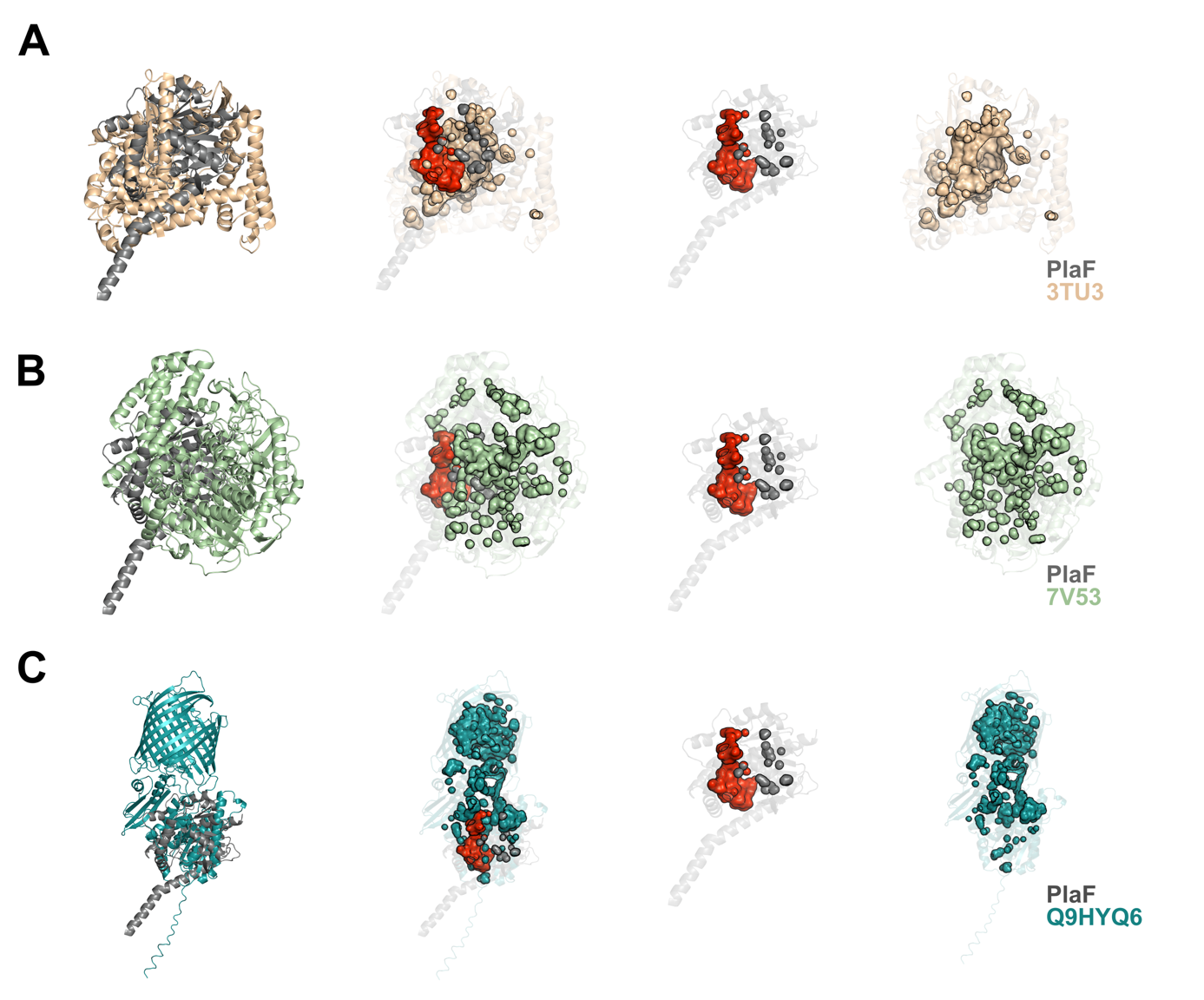


**Figure S9**. Structural superimposition of PlaF (grey) with ExoU (RCSB_ID: 3TU3) (pink) (A), PldA (RCSB_ID: 7V53) (green) (B), and OMPLA-PlpD (RCSB_ID: 5FQU, full sequence AlphaFold model AF-Q9HYQ6-F1) (turquoise) (C). Cavities within three solvent radii are shown as surfaces. The GW4869 cavity in PlaF is colored in red, the cavities in the other targets are colored according to the cartoon representation. The binding site cavity does not overlap with any of the suggested targets, suggesting high structural diversity.

**References**

1. Gentile R, Modric M, Thiele B, Jaeger K-E, Kovacic F, Schott-Verdugo S, Gohlke H. 2024. Molecular mechanisms underlying medium-chain free fatty acid-regulated activity of the phospholipase PlaF from *Pseudomonas aeruginosa*. Journal of the American Chemical Society Au 4:958-973.

2. Altschul SF, Gish W, Miller W, Myers EW, Lipman DJ. 1990. Basic local alignment search tool. Journal of Molecular Biology 215:403-410.
